# Insulators Target Active Genes to Transcription Factories and Polycomb-Repressed Genes to Polycomb Bodies

**DOI:** 10.1371/journal.pgen.1003436

**Published:** 2013-04-18

**Authors:** Hua-Bing Li, Katsuhito Ohno, Hongxing Gui, Vincenzo Pirrotta

**Affiliations:** Department of Molecular Biology and Biochemistry, Rutgers University, Piscataway, New Jersey, United States of America; New York University, United States of America

## Abstract

Polycomb bodies are foci of Polycomb proteins in which different Polycomb target genes are thought to co-localize in the nucleus, looping out from their chromosomal context. We have shown previously that insulators, not Polycomb response elements (PREs), mediate associations among Polycomb Group (PcG) targets to form Polycomb bodies. Here we use live imaging and 3C interactions to show that transgenes containing PREs and endogenous PcG-regulated genes are targeted by insulator proteins to different nuclear structures depending on their state of activity. When two genes are repressed, they co-localize in Polycomb bodies. When both are active, they are targeted to transcription factories in a fashion dependent on Trithorax and enhancer specificity as well as the insulator protein CTCF. In the absence of CTCF, assembly of Polycomb bodies is essentially reduced to those representing genomic clusters of Polycomb target genes. The critical role of Trithorax suggests that stable association with a specialized transcription factory underlies the cellular memory of the active state.

## Author Summary

We have studied the nuclear localization of genes that are regulated by Polycomb mechanisms. The genomes of higher eukaryotes contain hundreds of genes that are regulated by Polycomb mechanisms. Once repressed by Polycomb complexes, they tend to stay repressed; but, when activated, they bind Trithorax protein and tend to maintain the active state epigenetically. Polycomb repression has been reported to make these genes associate in the nucleus to form “Polycomb bodies.” We find that this association is not caused by Polycomb complexes but by insulator elements accompanying the genes. We show that, when these genes are in the active state, the binding of Trithorax targets them to other nuclear regions where transcription occurs, so-called transcription factories. In these nuclear re-positionings the insulator provides the associative power while the state of activity determines whether a gene goes to a Polycomb body or to a transcription factory. The strong effect of Trithorax suggests the possibility that the stable association with a transcription factory it produces may account for the epigenetic memory of the active state.

## Introduction

The default state for the organization of genomic material is the chromosomal territory occupied by the folding of the continuous chromatin fiber constituting a chromosome. From this territory, individual regions may loop out to partake in molecular activities such as transcription, heterochromatic silencing, Polycomb repression, etc. A question debated in the past few years is whether these different chromatin states are physically partitioned in the nuclear volume by targeting transcriptional activity to a transcription factory [1], [2], Polycomb repression to a Polycomb body [3], [4], etc. Is a chromatin domain that binds Polycomb group (PcG) proteins (or becomes transcriptionally activated) directed to a nuclear volume containing other PcG-repressed chromatin regions (or transcriptionally active genes)? Here we present evidence indicating that insulator elements play a crucial role in the formation of transcription factories as well as of Polycomb bodies.

Insulator-binding proteins organize the genomic material by forming networks of chromatin loops that govern both local higher-order folding and distant interactions between remote genomic sites [5], [6]. Insulator proteins, including CTCF in mammals, dCTCF, CP190, Su(Hw) and BEAF in *Drosophila*, have been mapped at thousands of sites throughout the genome that differ little from one cell type to another [7]–[9]. Although it is far from clear what fraction of these have enhancer-blocking function, most gene neighborhoods have at least one insulator, which could, in principle, govern the interaction of those genes with other genomic sites.

Polycomb bodies have been observed in mammalian and *Drosophila* nuclei by immuno-staining with antibodies against PcG proteins. Interactions between PcG target genes have been detected by 4C or Hi-C approaches [10], [11]. Genes residing in two different *Hox* gene clusters in *Drosophila* co-localize at significant frequencies within the same Polycomb body, resulting in enhanced silencing of both genes [12]. *Fab-7* and *Mcp* are so-called boundary elements that separate cis-regulatory regions of the Bithorax Complex in *Drosophila*. Each has been shown to contain two separable functional parts: a core Polycomb Response Element (PRE) and an insulator [13]–[16]. Transgenes containing the full *Fab-7* boundary region can interact with the endogenous *Fab-7* and co-localize at relatively high frequencies inside Polycomb bodies when both are repressed by PcG proteins [3], [4].

Using constructs containing the *bxd* PRE, *Fab-7* and *Mcp* elements, we have shown [17] that transgenes containing a PRE alone have no intrinsic ability to co-localize and that the insulator element, not the PRE, is necessary and sufficient to mediate long-distance interactions between *Fab-7* or *Mcp* transgenes. A comparison of our results with previous work [18], suggested that the addition of an enhancer might strongly increase co-localization. We show here that transcriptional competence is a major factor targeting co-localization: insulators target repressed genes to Polycomb bodies but also direct derepressed Polycomb target genes to foci of transcriptional activity. These associations are different from the local (1–3 Mb) interactions abundantly detected by genome-wide 3C-related approaches and can occur between different chromosomes. Although the interactions require “insulators” they are not constitutive: while insulator protein binding changes little, interactions occur only between genes in similar chromatin states, either both repressed or both active. We find that Trithorax is required for a stronger and stabler association of the derepressed gene to specific transcription factories, raising the possibility that the Trithorax-mediated epigenetic memory may owe more to nuclear localization than to histone modifications.

## Results

### The eye enhancer mediates high frequency co-localization in the eye disc

**Figure 1:**
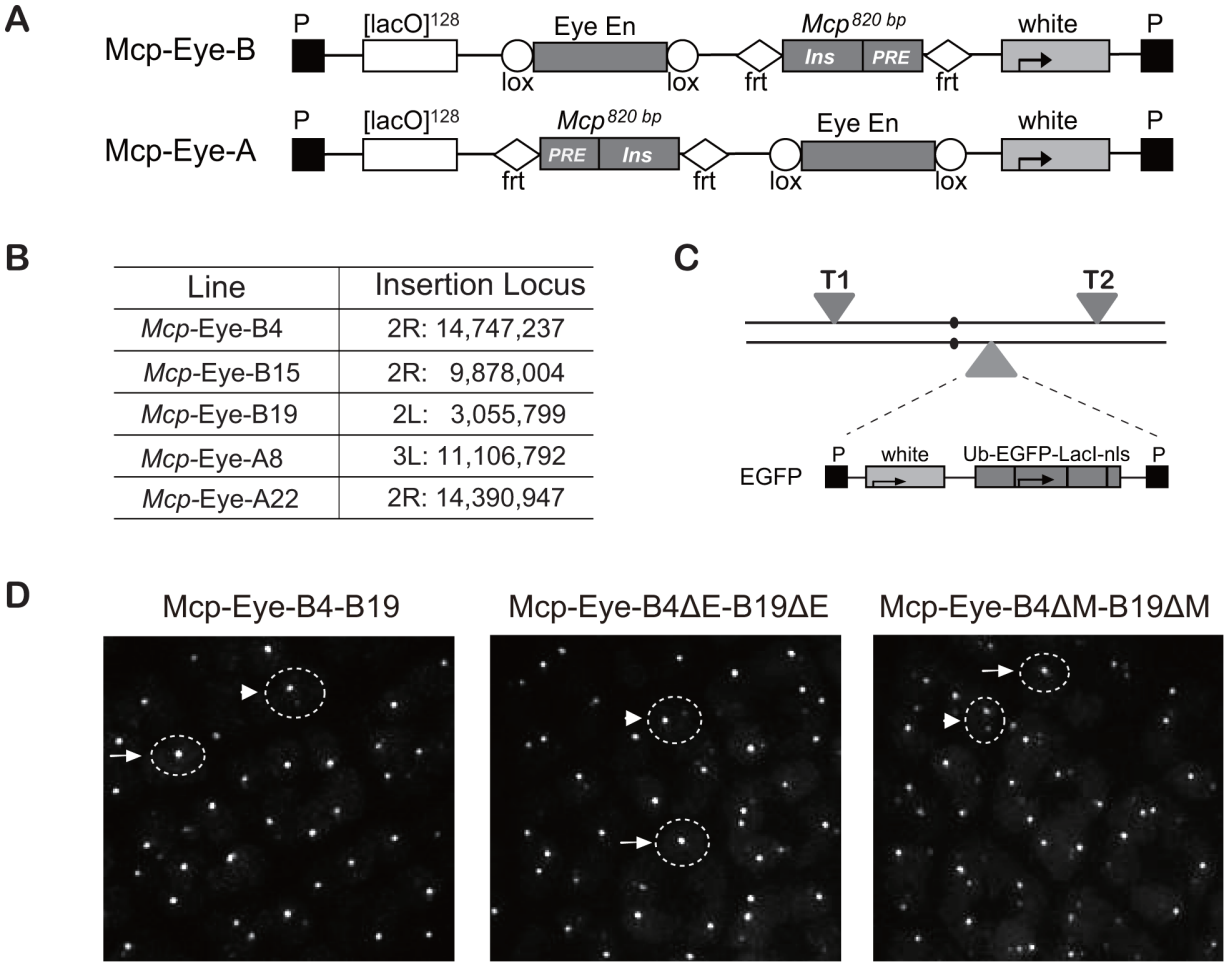
Transgene constructs to determine the effect of the Eye enhancer on co-localization. (A) Structure of the *Mcp*-Eye-B and A constructs. The *Mcp* 820 bp fragment contains the insulator and the PRE. The two constructs differ in the relative orientation and position of the Eye enhancer and *Mcp* fragments, flanked by Lox and FRT sequences, respectively, to allow excision. (B) Insertion sites of the *Mcp*-Eye-A and B transgene constructs with coordinates according to Release 5 of the Berkeley Drosophila Genome Project (http://www.fruitfly.org/sequence/release5genomic.shtml). (C) Genetic configuration for visualizing co-localization. Two transgenes T1 and T2 were typically recombined on the same or on different chromosomes while the homologue carried the construct expressing EGFP-*LacI* driven by the ubiquitin promoter (Ub). (D) Representative images from the eye imaginal disc. The dashed circles are approximate outlines of the nuclei as indicated by the background of EGFP targeted by a nuclear localization signal. A single bright spot (after scanning the nucleus in the z-axis) was taken to mean co-localization (examples indicated by arrows), while two (weaker) spots were interpreted as no co-localization (arrowheads). See also Video S1 and Figure S1.

Using tagged transgenes, we have previously shown that two copies of a *Mcp* transgene inserted at remote sites can physically co-localize in the nucleus. Constructs containing a minimal *Mcp* element associated in ∼7% of the nuclei in both eye and wing imaginal disc cells, dropping to less than 0.5% when the insulator part of *Mcp* was deleted [17]. To test the effect of transcriptional activation, we used the eye-specific enhancer of the *white* gene, active in the photoreceptor and pigment cells of the eye imaginal disc but not in the wing disc. The *Mcp*-Eye-B and *Mcp*-Eye-A constructs contain the 820-bp *Mcp* and the eye enhancer, flanked by FRT and Lox respectively, but with different position and orientation relative to the *white* reporter gene (Figure 1A). It is important to bear in mind here that, in the *Mcp* element, the PRE and the insulator have relatively weak effects as silencer or enhancer blocker, respectively [15]. The transgenes include 128 tandem *LacO* repeats, which are visualized in live cells expressing EGFP-*LacI* driven by the Ubiquitin promoter [17]. Previous experiments have shown that the vector itself, the FRT and Lox sites cause no interactions or specific localization effects [17], [18]. Three independent lines were used for *Mcp*-Eye-B and two for *Mcp*-Eye-A (Figure 1B and Figure S1).

**Figure 2:**
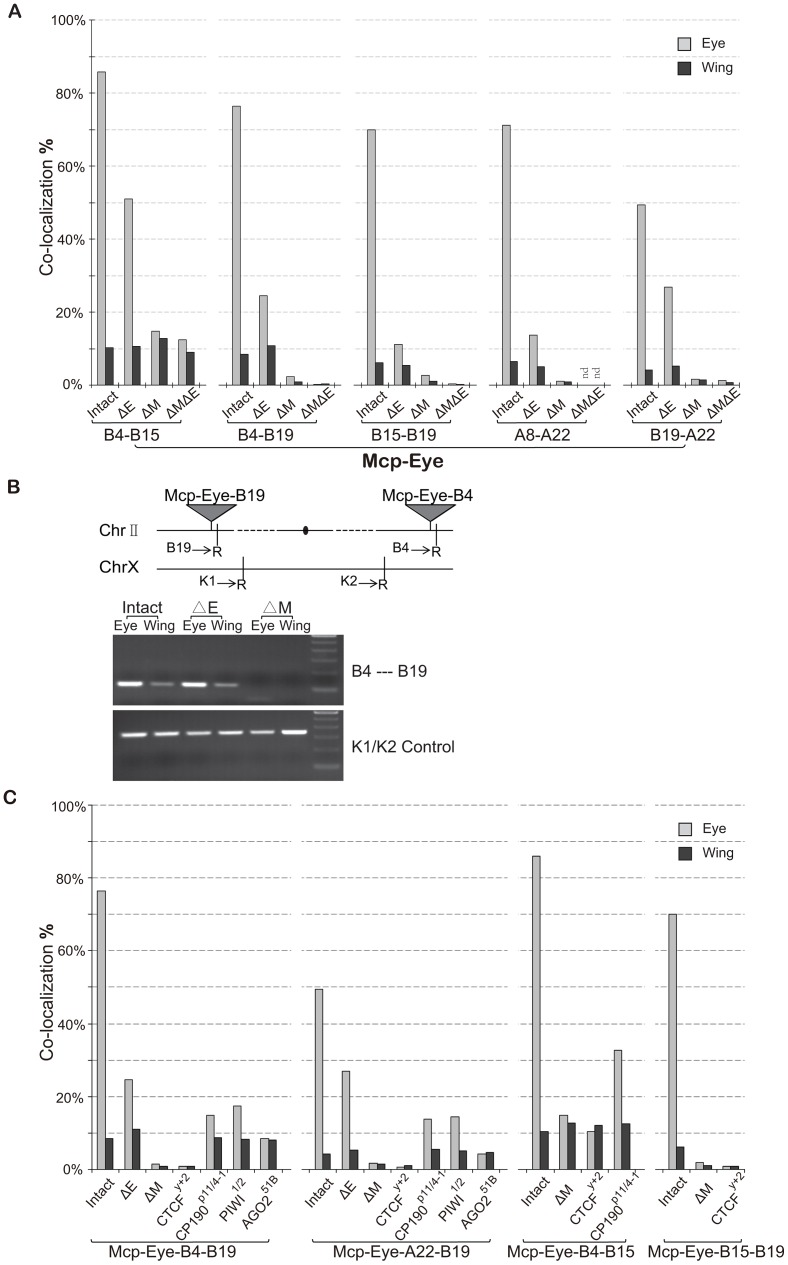
The eye enhancer greatly increases transgene co-localization. (A) Co-localization frequencies for *Mcp*-Eye transgene pairs. Gray bars: eye disc; black bars: wing disc. The Eye enhancer promotes high level co-localization in the eye but not in the wing disc, where it remains at the basal level. Excision of *Mcp* (ΔM) reduces co-localization to background level (<1%). Excision of the Eye enhancer (ΔE) reduces the frequency in the eye but not completely to the basal level. More than 500 nuclei (N>500) were counted for each individual line and the χ^2^ test gives p-values <0.0001 that the differences might be due to chance in all cases (Table S2). (B) 3C assays confirm the co-localization results in wing and eye imaginal discs separately dissected from *Mcp*-Eye-B4–B19 larvae or their ΔE and Δ*Mcp* derivatives. The schematic map shows the two transgene insertions on chromosome 2. The gel picture, corresponding to the B4–B19 data in Figure 2A, shows the PCR products using primers (arrows) for genomic sequences adjacent to *EcoR*1 sites (R) flanking the transgene insertion sites. The lower gel shows internal controls using K1 and K2 primers for ligation of two adjacent *EcoR*I fragments. The endpoint PCR analysis is less sensitive to differences in co-localization than the imaging approach and does not reveal the effect of enhancer loss. (C) Effects of insulator protein mutations. Co-localization frequencies are shown for wing and eye disc from four *Mcp*-Eye transgene combinations. Loss of CTCF has the same effect as excision of *Mcp*: co-localization drops to background level (<1%). Loss of CP190 or of RNAi components *piwi* and *AGO2* affects the high-level co-localization but not the basal level (see also Figure S3). χ^2^ test gives p-values <0.0001 in all cases (Table S2). See also Figure S1, Figure S2, and Table S2.

We combined two transgene insertions into a fly line that also expressed EGFP-*LacI* (Figure 1C), and visualized the interaction between the two transgenes by live imaging of eye and wing imaginal discs. In the reconstituted 3D images (Figure 1D and Video S1), nuclei with one dot were taken to indicate co-localization and two dots as no interaction (see Methods section and [17], [18]). In all combinations, we found that the 820-bp *Mcp* mediated high frequency interactions (50–90%) in the eye imaginal disc cells, but low co-localization (5–10%) in wing disc or in the cells of the membrane surrounding the eye disc (Figure 2A). The combination of two A transgenes, or two B transgenes, or an A and a B transgene, all give similar co-localization frequencies, irrespective of the different relative position and orientation of the *Mcp* and enhancer. This and the eye colors of the flies (Figure S1) are consistent with the idea that the enhancer-blocking activity is weak. The orientation does affect pairing-dependent silencing: all the *Mcp*-Eye-B lines show pairing-dependence while the *Mcp*-Eye-A lines (in which the *Mcp* insulator is interposed between the PRE and the promoter) are not pairing-sensitive (Figure S1).

These results suggest that, surprisingly, transcriptional activation greatly increases the association of two *Mcp* transgenes in the nucleus. To demonstrate this, we deleted the eye enhancer (ΔE), using the Cre recombinase. This dramatically decreases the frequency of co-localization in the eye disc cells in all five combinations (from ∼70% down to ∼20%; χ^2^ test, p<0.0001) (Figure 2A). Similar decreases result when the enhancer is deleted from only one of the two transgenes (Figure S1). Without enhancer, co-localization remained consistently higher in eye disc cells (∼20%) than in wing and membrane cells (4–10%), probably because the reporter *white* gene has residual eye-specific activity, as evidenced by the eye colors. We conclude that enhancer activity helps to bring two distant transgenes together in the eye cells, presumably in a different nuclear environment from that of the repressed genes. Rather than in a Polycomb body, we suppose that the site of activity will be a transcription factory. In the absence of the enhancer, some association will persist but at different nuclear locations either when both genes are active from residual eye-specific activity (transcription factory) or when both are Polycomb-repressed (Polycomb body).

### Insulators are required for long-range interaction

Next, we deleted the *Mcp* part of the transgene (ΔM) to see if it is necessary to mediate the long-range interaction. The results (Figure 2A) show that co-localization is almost totally abolished both in eye and wing imaginal disc cells (from 70% to <2%; χ^2^ test p<0.0001). Double deletion of both *Mcp* and Eye enhancer gives similar results (Figure 2A, ΔMΔE) except in the case of the *Mcp*-Eye-B4-B15 pair, probably because the two transgenes are fairly close to each other (∼5 Mb) on the same chromosome arm. Therefore enhancer-dependent transcriptional activity is not sufficient to promote long-range interactions in the absence of the Mcp insulator+PRE.

We also carried out a 3C assay with one of the combinations (*Mcp*-Eye-B4–*Mcp*-Eye-B19) to show that the B4 and B19 transgenes, inserted on two different arms of chromosome 2, interact physically with one another in the eye disc but not in the wing disc. This interaction is still strong after enhancer deletion, but disappears after *Mcp* is deleted from one of the two transgenes (Figure 2B).

Excision of the *Mcp* fragment removes both insulator and PRE functions. The *Mcp* insulator binds the insulator proteins CTCF and CP190 [8], [17], [19], [20] and is required for co-localization [17]. To determine if the insulator is still specifically required for the high level co-localization, we made the flies homozygous for the loss of function mutation *CTCF* ^y+2^. The loss of CTCF reduces the interaction of two remote transgenes to a level similar to that seen when *Mcp* is deleted (Figure 2C). CP190 mutations have a weaker effect in the eye disc (from 70% to ∼14%; p<0.0001), but do not alter interaction frequencies in the wing disc or in the eye disc membrane cells, where the eye enhancer is not active. We conclude that insulator function is still essential for long-range interaction and, in its absence, the enhancer alone cannot promote interaction. The CTCF protein is required for this insulator function, while CP190 contributes to the high frequency interaction but not to enhancer-independent interaction.

RNAi components interact with insulator proteins [21], [22] and have been implicated in the *Fab-7*-mediated long-distance interactions [4]. The interactions of our *Mcp* transgenes are also affected by mutations in the RNAi genes *piwi*, *aub* and particularly *AGO2* (Figure 2C and Figure S2). Like CP190 mutations, RNAi mutations reduce the high level co-localization in the eye disc without affecting that in wing cells. As previously reported [22], the role of AGO2 does not require its catalytic activity since the AGO2-V966M mutation, which abolishes catalytic function, has no effect (Figure S2).

In human cells, cohesin proteins co-localize extensively with the insulator protein CTCF and, together with CTCF, mediate long-range interactions [23], [24]. In *Drosophila*, ChIP data do not show such a relationship and the genetic evidence does not support it. We found that loss of function of *Smc1* or *Rad21* does not affect the co-localization frequency in eye or wing disc cells (Figure S2) although it does abolish pairing-dependent silencing effects (results not shown).

### TRX but not PC is required for high-level interaction

**Figure 3:**
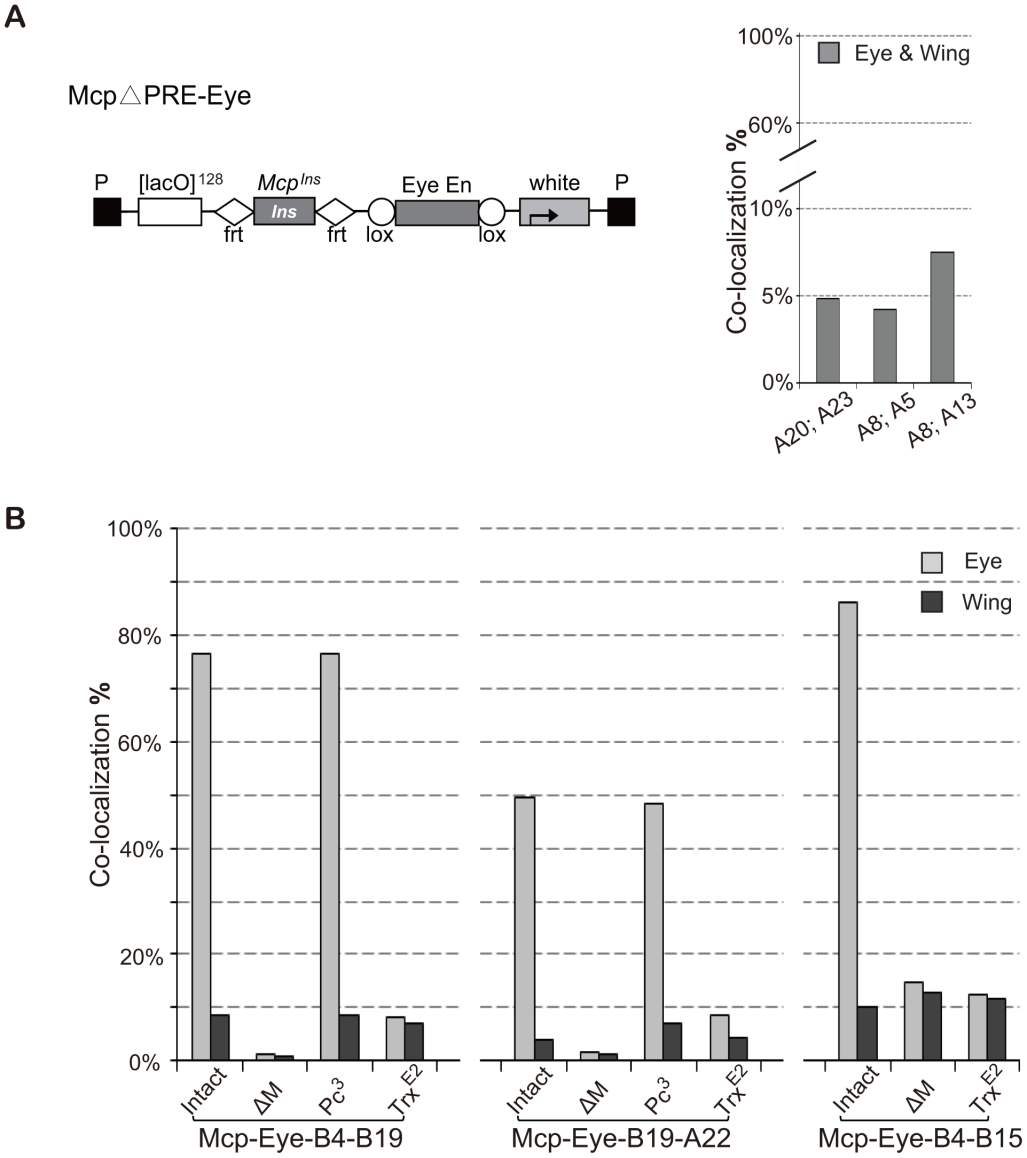
High frequency co-localization to transcription factories depends on Trithorax. (A) Map of *Mcp*ΔPRE-Eye. The *Mcp* insulator is flanked by FRT sequences and the Eye Enhancer by Lox sequences to allow excision. Note: this construct has no *Mcp* PRE. Lines A20 and A23 are inserted on the X chromosome; A5 and A13 on chromosome 3; A8 on chromosome 2. Co-localization between pairs of *Mcp*ΔPRE-Eye insertions does not rise above the basal level, suggesting that the *Mcp* PRE is necessary for high frequency. (B) Effects of *Pc* and *trx* mutations on co-localization of *Mcp*-Eye. Pairs of *Mcp*-Eye transgene insertions were tested with *Mcp* (intact), with *Mcp* deleted (ΔM) or in the presence of heterozygous *Pc* ^3^ or *trx* ^E2^ mutations. While *Pc* has little effect, halving the TRX dosage reduces co-localization to the basal level. χ^2^ test gives p-values of 0.9913 for B4–B19 *Pc* ^3^ and 0.674 for A22–B19 *Pc* ^3^. In all other cases p-value <0.0001 (Table S2). See also Figure S3 and Table S2.

Since PREs are generally repressive, we reasoned that the high interaction we observed might only need the insulator part of *Mcp* plus the enhancer activity. To test this, we constructed *Mcp*ΔPRE-Eye, similar to *Mcp*-Eye-A but containing only the insulator part of *Mcp* instead of the 820-bp *Mcp* (insulator+PRE) fragment (Figure 3A). Unexpectedly, several combinations of *Mcp*ΔPRE-Eye insertions show only the basal 5∼7% insulator-dependent co-localization frequency both in eye and wing disc cells (Figure 3A), similar to that obtained with the insulator alone, with no enhancer or PRE [17]. These results indicate that enhancer-promoted transcriptional activity is not sufficient and that the PRE is in fact important for the enhancer to mediate high frequency long-range interactions.

To understand the role played by the PRE, we returned to the 820-bp *Mcp*-Eye lines, and tested them in a *Polycomb* mutant background. Since homozygous *Pc^−^* flies die at the embryonic stage, we tested heterozygous *Pc^−^* larvae and found that halving *Pc* dosage, which often has a detectable effect on the expression of a PcG-repressed transgene, has no effect on the long-distance interaction (Figure 3B). High-level interaction is therefore not sensitive to PC levels, as expected since PC generally does not bind when the gene is in the active state (Figure S3) [25].

PcG target genes are positively regulated by Trithorax (TRX), a histone methyltranferase homologous to mammalian MLL1, known to methylate H3K4 and to antagonize PcG repression [26]–[30]. TRX binds constitutively to all known or putative PREs (therefore also called TREs) regardless of whether they also bind PcG proteins or whether the target genes are transcriptionally repressed [25]. To test whether TRX is required for high level co-localization, we crossed our *Mcp*-Eye lines into a *trx* mutant background. Homozygous *trx* loss of function mutations are embryonic lethal but, even in heterozygous *trx* larvae, we found that the high co-localization in the eye disc cells is reduced to the basal level, the same level found in eye membrane and wing disc cells (Figure 3B). Together, these results demonstrate that the high frequency co-localization of the active transgenes is highly dependent on TRX concentration but not on PC concentration and therefore requires TRX/TRE but not PC/PRE function.

### Other enhancers also promote co-localization

**Figure 4:**
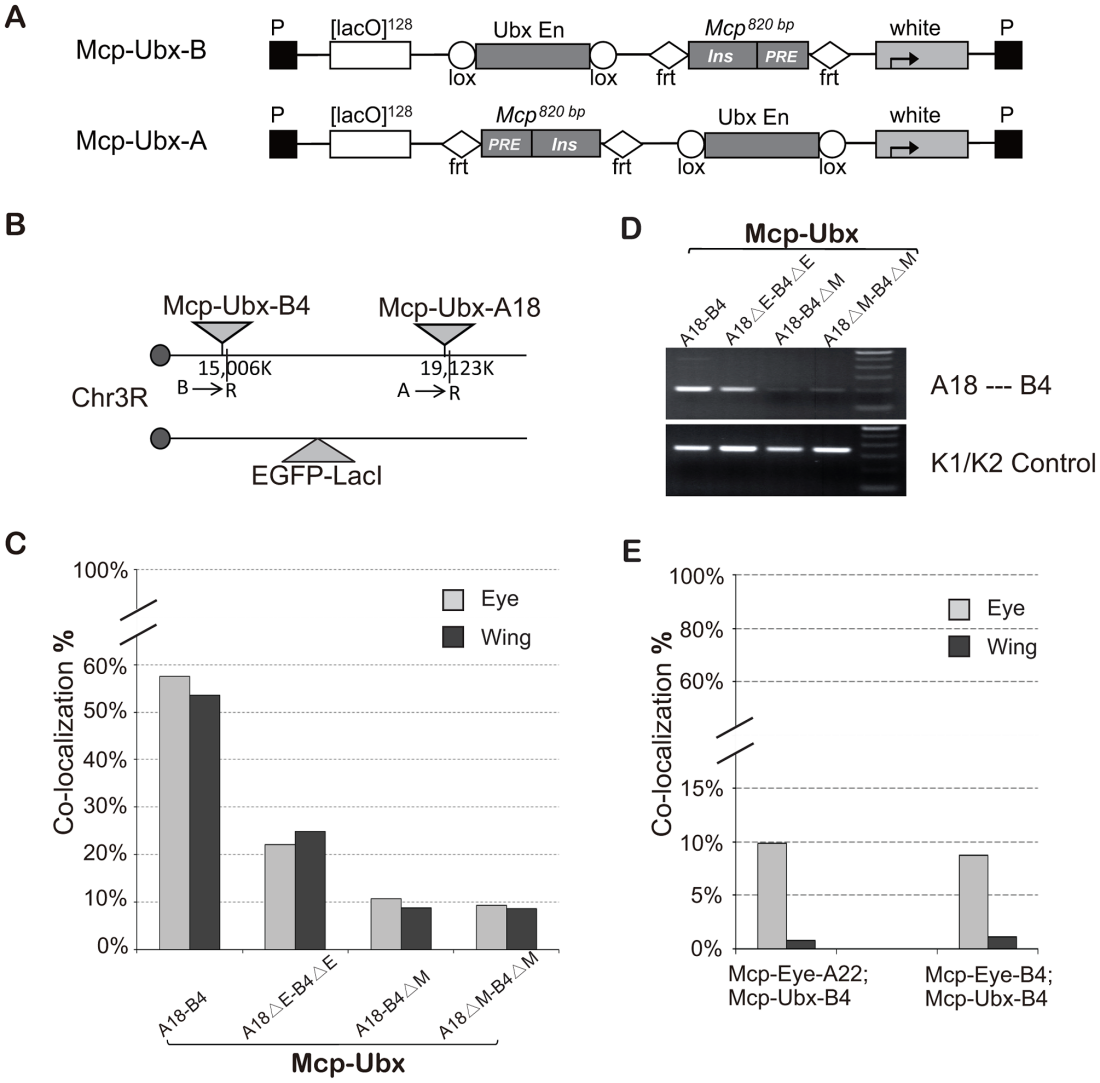
A *Ubx* enhancer also promotes high level co-localization. (A) Map of *Mcp*-*Ubx* transgenes, containing the *Ubx* imaginal disc enhancer. (B) Schematic representation of the two *Mcp-Ubx* transgenes tested for co-localization. (C) Frequencies of co-localization of the *Mcp*-*Ubx* transgenes in (B). Since the *Ubx* H1 imaginal enhancer is active in both eye and wing discs, the two have similar co-localization frequency (∼55%). Enhancer excision (ΔE) reduces co-localization to the basal level, relatively high here (23%) because the two transgenes are only 4.1 Mbp apart. Similarly, the background level upon excision of one or both *Mcp*s is relatively high (10%). The two-tail paired *t* test gives p-value = 0.535 between eye and wing discs. The χ^2^ test gives p-values <0.0001 in cases of enhancer deletion and *Mcp* deletion. (D) 3C analysis of the *Mcp-Ubx* A18—B4 interaction. The analysis was done with combined eye and wing discs from the same fly lines used in Figure 4C and shows a decrease in interaction after deletion of the enhancers but loss of interaction when one or both Mcp are deleted. (E) Co-localization between *Mcp*-Eye and *Mcp*-*Ubx*. Basal co-localization occurs in the eye disc, where both transgenes are active but under different enhancers. Background levels are seen in the wing disc where one transgene is active and the other silent. χ^2^ test gives p-values <0.0001 that differences are not due to chance in all cases in (C) and (E) (Table S2). See also Figure S4 and Table S2.

To test if a different enhancer can also promote the long-range interaction mediated by the insulator, we constructed two new transgenes, *Mcp*-*Ubx*-B and *Mcp*-*Ubx*-A (Figure 4A), in which the eye enhancer in *Mcp*-Eye is replaced by the *Ubx* H1 enhancer, which drives strong and uniform expression in eye, wing and haltere imaginal discs [31]. We recovered two independent *Mcp*-*Ubx* insertions, both on the right arm of chromosome 3 (Figure 4B). After combining the two insertions into one line, robust high-level co-localization (∼55%) was observed in all the cells of both eye and wing discs, showing that wing cells are not intrinsically less suited for co-localization than eye cells (Figure 4C, t test, p = 0.535). Co-localization decreased to a moderate level (23%) after deletion of the *Ubx* H1 enhancer, and down to background level (∼9%: the two transgenes are only 4.1 Mb apart) after deletion of one or both *Mcp*s (χ^2^ test, p<0.0001). The 3C assay was also used to confirm that the interaction between the two transgenes decreases after enhancer deletion and is not detected after Mcp deletion from one or both transgenes (Figure 4D). We conclude that different enhancers can promote co-localization mediated by *Mcp*. Similar but weaker results were obtained with *Mcp*-UAS, in which the eye enhancer of *Mcp*-Eye-A was replaced by five copies of the GAL4 binding site (5×UAS) and activated by the *arm*-GAL4 driver (Figure S4).

### High-frequency co-localization may require shared enhancer factors

To look at interactions between transgenes containing different enhancers we used combinations of *Mcp*-Eye (eye enhancer, active in the eye) and *Mcp-Ubx* (*Ubx* H1 enhancer, active in eye and wing), both inserted in chromosome 3. As shown in Figure 4E the two transgenes do not co-localize in wing cells where one is active and the other silent, and have the normal level (∼10%) but not the high level of co-localization in the eye, where both are active. We suppose that when the *Mcp*-Eye and *Mcp*-*Ubx* transgenes are in different transcription states, they are directed into different nuclear domains. When both transgenes are active, the fact that they interact only at the basal level suggests that, while they may chance to land in the same active compartment, they lack high frequency co-localization, which probably requires sharing transcriptional activators.

### Active transgenes interact with active endogenous homeotic genes

So far, our results have shown that two *Mcp*-containing transgenes co-localize with one another when they are both repressed and, much more frequently, when they are both activated by the same enhancer. In an earlier paper we showed that they can also interact with the endogenous *Mcp* element [17]. The endogenous *Antp* gene of ANT-C has been reported to co-localize in a Polycomb body with the *Abd-B* gene of the Bithorax Complex (BX-C) when both genes are PcG-silenced, but not when one is active and the other silenced [12]. Next we asked if similar rules govern interactions between a *Mcp* transgene and other endogenous *Hox* genes. Since the eye enhancer is active only in a subset of cells of the eye-antenna disc, we turned to the *Mcp*-*Ubx* constructs containing the *Ubx* imaginal enhancer, active in all wing and eye disc cells, as are also ANT-C genes, unless repressed.

**Figure 5:**
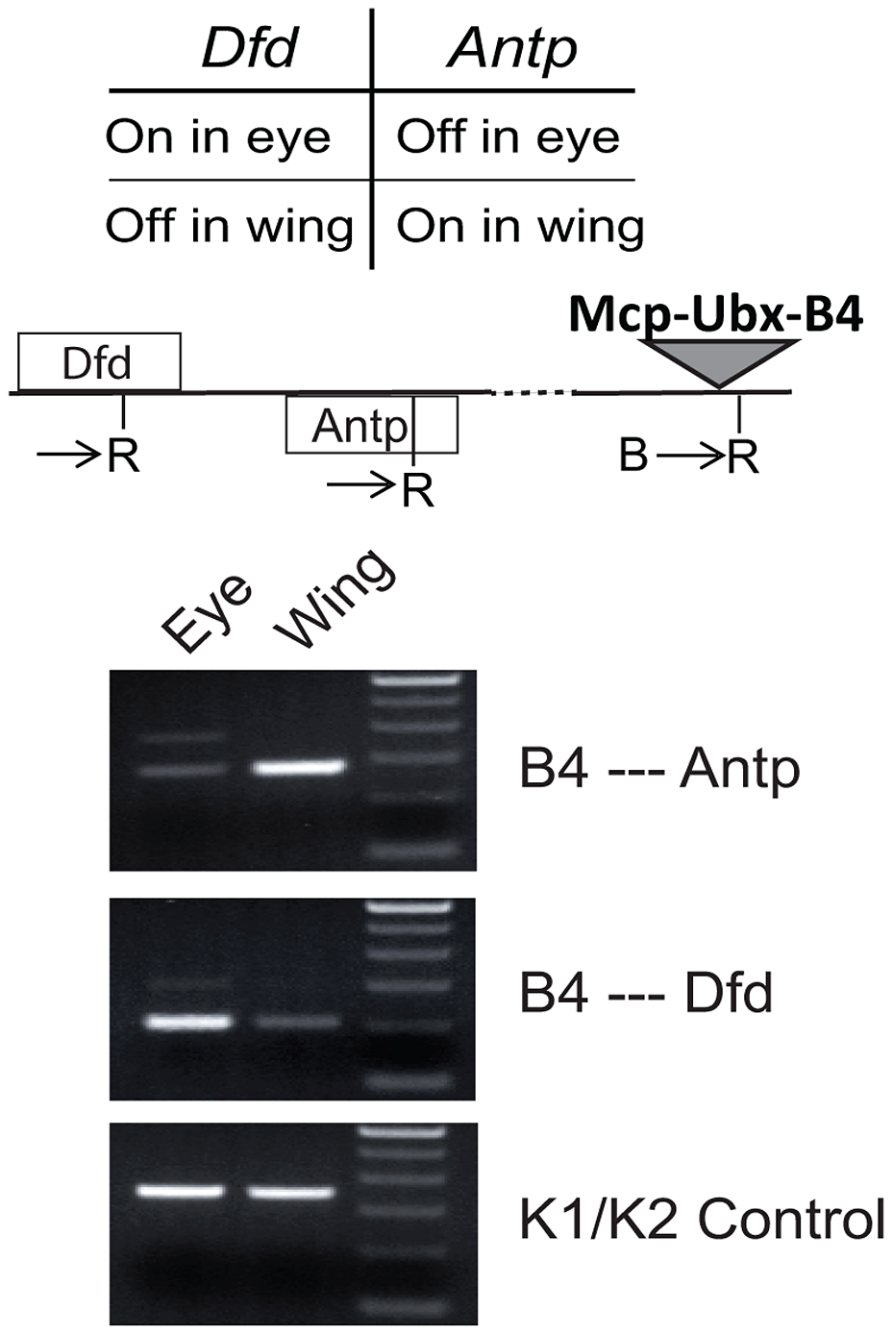
An active transgene interacts only with active endogenous homeotic genes. The B4 *Mcp-Ubx* transgene interacts with an endogenous *Hox* gene when both are active. The map shows the positions of the *Dfd* and *Antp* genes, both in the ANT-C and the *Mcp-Ubx* transgene. The PCR primers used are indicated, relative to a flanking *EcoR*I site (R). The 3C PCR reactions show that the transgene, which is active in both eye and wing discs, interacts significantly with the *Hox* genes only when they are active.

To determine associations with endogenous genes, we used 3C analysis of the *Mcp*-*Ubx* line containing an intact transgene Mcp-Ubx B4 (Figure 4A). We tested the interaction of our transgene with *Antp* and *Dfd*, both *Hox* genes belonging to the ANT-C. *Antp* is active in the wing and silenced in the eye, while *Dfd* is silenced in the wing and active in the eye. To determine the interactions in the different combinations of states, we isolated eye imaginal discs separately from wing discs of the intact *Mcp*-*Ubx* line and performed the 3C assay on each. As shown in Figure 5, the active transgene *Mcp*-*Ubx*-B4 interacts only with active homeotic genes, specifically, with *Antp* in the wing and with *Dfd* in the eye disc cells.

### Association of endogenous PcG target genes also depends on CTCF and TRX

**Figure 6:**
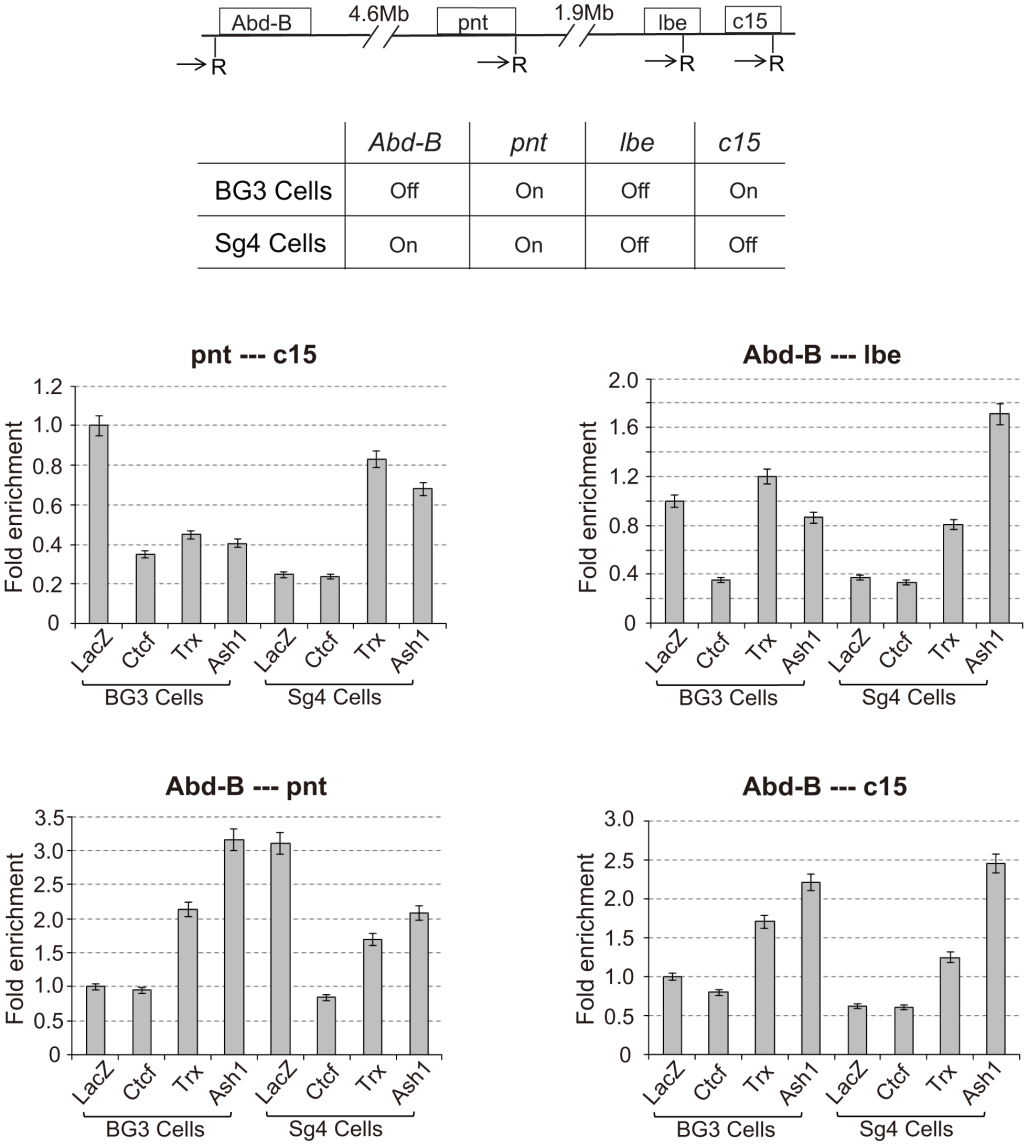
Interaction between endogenous PcG target genes is dependent on CTCF and TRX. The upper panel is a schematic map showing the location of four endogenous PcG target genes on chromosome 3R. The PCR primers flanking *EcoR*I sites used for 3C are indicated by arrows. The middle panel shows the transcriptional status of the four genes in the BG3 and Sg4 cell lines [25]. In the lower panels, the 3C PCR reactions, quantified by qPCR, standardized in each case to the control ligation of adjacent fragments using K1 and K2 primers, are reported relative to the value of control BG3 cells (LacZ). The cells were treated respectively with RNAi against LacZ (control), CTCF, *trx*, or *ash1*. The results show that two PcG genes interact with each other only when both are active or both are silenced, but not when one is active and the other is silenced. All interactions are affected after CTCF RNAi; *trx* and *ash1* knock-downs affect only interactions between two active genes. See also Figure S5.

The associations detected with the various *Mcp* transgenes are not a peculiarity of *Mcp* or of the transgene constructs. We tested endogenous PcG target genes *Abd-B*, *pnt*, *lbe* and *C15*, all on chromosome 3R, for their ability to associate in the repressed or active state using the 3C approach. We used two different cultured cell lines, BG3 and Sg4, in which some of the genes are in different states of activity, and we treated the cells with RNAi against CTCF, *trx*, *ash1* or *lacZ* as a control (see Figure S5). The 3C products were analysed by qPCR standardized in each case by the products for the ligation between adjacent control fragments and are shown in Figure 6 in terms of fold enrichment relative to the result in BG3 cells (LacZ control RNAi). The first panel displays the results for interactions between *pnt* and *C15*. These genes are both active in BG3 cells and show significant interaction. The interaction decreases upon knockdown of CTCF, TRX or ASH1. In Sg4 cells *pnt* is still active but *C15* is off. The interaction in control Sg4 cells (LacZ RNAi) is greatly decreased relative to that in BG3 cells. It is equally low upon CTCF knockdown but increases when TRX or ASH1 are knocked down: in their absence the *pnt* gene is again repressed by Polycomb and is able to interact with the repressed *C15* gene. Interactions between *Abd-B* and *lbe* follow a similar pattern: in BG3 cells where both are PcG-repressed they show CTCF-dependent interaction not affected by *trx* or *ash1* knockdown. They do not interact in Sg4 cells where one is active and the other repressed but, in the absence of TRX or ASH1, we presume that the active gene becomes at least partially repressed and interacts with the repressed gene. These interactions are all between different genes with different enhancers and are therefore analogous to those represented in Figure 4E and Figure 5. Overall, the four panels show that interactions are high between genes when they are both PcG-repressed or both in the active state but decrease when one is active and one repressed (Figure 6). All interactions are affected by CTCF RNAi. Knockdown of *trx* or ash1 affects interactions between active genes but not those between repressed genes.

**Figure 7:**
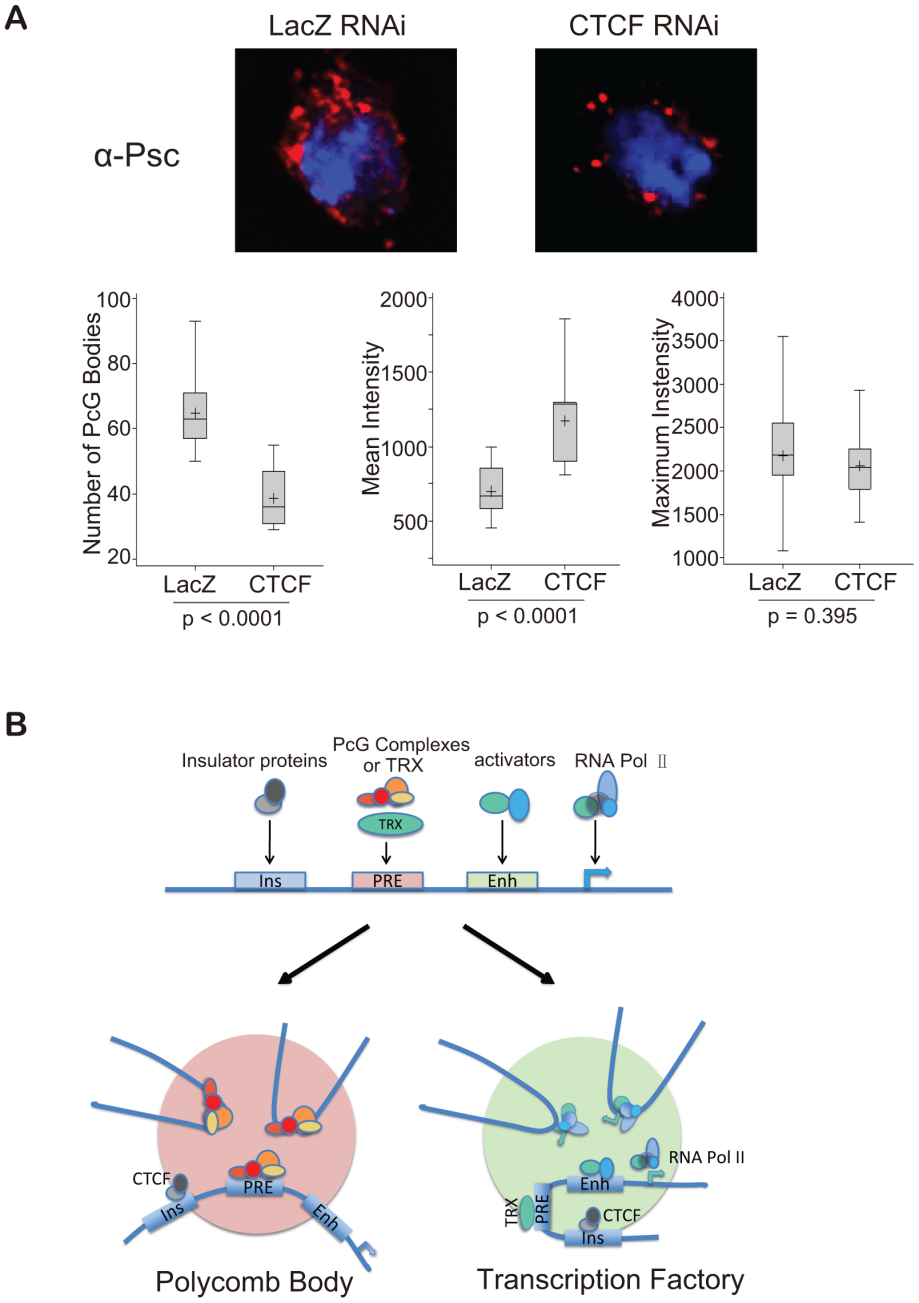
A comprehensive model for insulator-mediated nuclear localization. (A) CTCF RNAi reduces the number of Polycomb bodies. Polycomb bodies, visualized with anti-PSC (red), decrease in number and size, mostly losing the weaker foci. DNA was stained with DAPI (blue). To quantify the results, 11 nuclei of LacZ RNAi-treated BG3 cells and 15 nuclei of CTCF RNAi-treated cells were analysed with NIH ImageJ. The two sets of data were compared using the *t*-test, p values are shown below each box plot. The median is indicated by a line across the box, the mean value by “+”. (B) Model representing a generalized PcG target gene with PRE, enhancer (Enh) and insulator (Ins), as well as the promoter. When the gene is repressed by PcG complexes, CTCF, perhaps with suitable posttranslational modifications, targets it to Polycomb bodies (pink). When the gene is switched on, TRX becomes active, and the gene is targeted by CTCF, perhaps with activation-specific modifications, to transcription factories (green). See also Figures S5, S6, and S7.

These results imply that Polycomb bodies formed by the association of remote PcG targets fall apart in the absence of CTCF. Of course, a significant number of Polycomb bodies are structurally determined by the genomic clustering of many PcG target genes (this argument is developed in ref. 32). Egregious examples are the two Drosophila *Hox* clusters: the Antennapedia Complex (ANT-C) and the Bithorax Complex (BX-C). In the absence of CTCF the ANT-C genes would continue to be clustered and so would the BX-C genes but the two clusters would no longer associate (Figure S6). To see if this effect could be visualized, we immunostained the cells treated with CTCF RNAi with anti-PSC or with anti-RNA pol II to illuminate respectively Polycomb bodies (Figure 7A) or transcription factories (Figure S3). Compared to the cells treated with the control *lacZ* RNAi, the CTCF RNAi cells appear to have fewer and brighter Polycomb bodies suggesting that numerous smaller bodies have been lost. A quantitative analysis was made to determine the number of foci above threshold and their mean and maximum intensities (Figure 7A). The results confirm that, in the absence of CTCF, the number of visible foci decreases due to loss of the weaker foci. The high intensity foci are not affected. These are expected to be the structural clusters because they are associated 100% of the time while genes interacting through an insulator associate only part of the time. We therefore interpret these results as consistent with our expectation of the role of CTCF. The RNA pol II images are more difficult to evaluate but clearly many transcription factories have not dissociated and we cannot say whether CTCF plays a general role (Figure S7).

## Discussion

### Ground rules for nuclear localization

Some basic rules can be deduced from our results. A background level of interactions is proximity-dependent and insulator-independent. When two transgenes both contain an insulator, interaction frequency rises to a level of 5–20%. Interactions are more frequent between sites on the same chromosome arm, as has also been reported by others [10], [32] but they can be observed also between sites on different chromosomes [3], [32], [34]. Interactions beyond the chromosome arm have also been detected by genome-wide 3C methods but appear to be underrepresented in these approaches, probably because these methods are ligation-dependent and detection of individual interactions is in competition with that of the much more abundant proximity-dependent associations. Whether a PRE makes any contribution to the basal level is unclear but we detected none when we tested specifically for it [17]. The addition of an enhancer to both transgenes produces a dramatic increase in the frequency of co-localization (50–90%) that requires the presence of a PRE/TRE, as well as an insulator, and implies a remarkably stable association. The nature of the enhancer factors probably plays a role since co-localization between transgenes with different enhancers occurs in tissues where both are active but remains at the 5–20% level. In most cases, interactions between endogenous PcG target genes fall into this category: they interact when both are active or when both are PcG-repressed but not at the highest levels seen between genes activated by the same enhancer.

The results of a large number of experiments with different transgenes and different fly lines [17], [18], [32], [34] are consistent and are not attributable to the peculiarities of a few specific insertion sites although co-localization levels are probably influenced by the genomic environment. Our results show that these conclusions are also true for endogenous PcG target genes.

### Insulator function

Both CTCF and CP190 insulator proteins bind to the *Mcp* insulator. Loss of CTCF function has the same effect as deletion of the *Mcp* insulator, reducing co-localization to generally less than 1%, which we take to be the level due to chance encounters. It is highly unlikely that this effect is due to misregulation of some unknown gene that affects our transgenes because a) it mimics the effect of deleting the insulator, b) it affects both localization to Polycomb bodies and to transcription factories, c) similar effects are observed for several endogenous genes, d) genome-wide studies in cultured cells show that the loss of CTCF does not have major global effects on gene expression [35]. Surprisingly, loss of function CP190 mutations have a more nuanced effect suggesting that CP190 is only required for co-localization in the active state or that loss of CP190 does not abolish insulator action as completely as loss of CTCF. A major implication of these experiments is that co-localizations between different repressed PcG target genes [10], [11] are mediated by insulator-binding proteins rather than by interactions between bound PcG complexes. Earlier results indicate that the *gypsy* insulator, which binds SU(HW), CP190 and MOD(MDG4) proteins, has an effect similar to that of the *Mcp* insulator, which binds CTCF and CP190, on the interaction between remote transgenes [34].

CTCF function in mammalian genomes is tightly linked to cohesion [23], [36]. In *Drosophila*, however, there is no apparent relationship between cohesin and CTCF in chromatin binding or in insulator function. Consistently, co-localization is not affected by mutations in the major cohesin components Smc1 or Rad21 although these mutations abolish the pairing-dependent silencing effect (results not shown) seen typically with transgenes containing *Mcp* and other PREs [32], [37].

Lei and Corces [21] first reported that insulator functions are affected by mutations in the RNAi machinery. We find in fact that the co-localization is affected by the same mutations in the RNAi machinery. Here too, however, the effects are complex, with some RNAi proteins affecting high-level co-localization but not the basal level. These results suggest that the action of the insulator element involves an RNA component whose nature and role remain unknown. Interestingly, loss of AGO2 has the strongest effects on the high level enhancer-promoted co-localization but no effects on the basic co-localization in the wing disc and its role does not require its enzymatic activity, as previously reported for insulator function [22].

### The role of Trithorax

The powerful effect of TRX mutations, perhaps the most intriguing feature of the high-level co-localization, suggests that TRX provides an additional level of stability to the association with a common transcription factory. Loss of co-localization when TRX levels are reduced is not because the transgene is no longer active. Transcription is only slightly reduced, as shown by the eye color of the transgenic flies and by the expression of endogenous genes [29], [30]. When PcG target genes become transcriptionally active, they form a chromatin domain that binds the TRX N-ter moiety and ASH1 [25]. Genetic evidence shows that the role of these two proteins is to antagonize PcG repression and maintain a “cellular memory” of the derepressed state [29], [30], a role that is not shared by Set1, the methyltransferase that targets H3K4 in the promoter region of most active genes. The effect of TRX is therefore not likely to be due to H3K4 methylation. Although we do not yet understand the molecular bases of this epigenetic memory, the powerful effect of TRX suggests (but does not prove) that co-localization may play a part. It may be relevant to this role that MLL1, the mammalian TRX homologue, remains associated with promoters in mitotic chromosomes [38]. It is possible therefore that TRX and ASH1 mediate a more stable association of the derepressed gene with a transcription factory that specializes in TRX-dependent transcription and that the epigenetic memory is more dependent on nuclear localization than on the classical histone modifications.

### Targeting genes to nuclear domains

Together with recent work in flies and in mammals, our results indicate that insulator binding proteins have much broader functions than blocking inappropriate action of enhancers and silencers. Our results assign to them a key role in the association of genes repressed by the PcG machinery and in the congress of these genes when they are in the active state. We have not determined directly whether the genes tested are in Polycomb bodies or in transcription factories when they co-localize and, in fact, in any one nucleus a gene may be located in one compartment at one time and another compartment at a later time. However, we have shown that all co-localization of our transgenes is dependent on CTCF, which must therefore be responsible for targeting in both the repressed and active state. In the model shown in Figure 7B, the insulator protein targets a PcG target gene to a Polycomb body when the gene is PcG-repressed. The binding of activators to the enhancer switches the gene to the active mode characterized by the activation of TRX, recruitment of ASH1 [25] and targeting of the gene to a transcription factory. The role of TRX is crucial for high level targeting, implying that it makes a major contribution to the stability of the association with the transcription factory and suggesting that a subset of transcription factories specializes in TRX-dependent genes. We have not looked for a role of insulators in targeting to transcription factories genes that are not PcG targets.

How could the same insulator-binding proteins direct a gene to PcG bodies when it is PcG-repressed but to transcription factories when it is in the transcriptionally active state? Although our experiments give no answers to this question, we have proposed that insulator complexes might be post-translationally modified depending on adjacent silencing activity or transcriptional activity and that such modifications might select the appropriate nuclear compartment [33]. For example, sumoylation of mammalian CTCF has been reported and related to the SUMO-E3 ligase activity of PC2h [39], [40].

Is there a functional advantage to co-localization in either case? This is difficult to evaluate but the following arguments suggest that there is. PcG repression in Drosophila displays well-known pairing-dependent effects. A transgene containing a PRE that is partially repressed when present in one copy, usually becomes much more strongly repressed when the transgene insertion is homozygous [37]. Since homologous chromosomes are paired during interphase in Drosophila, this implies that physical proximity of two (or more) PREs increases the degree or stability of silencing. In the case of an active gene, it has been shown that a PcG target gene such as *Ubx* is more strongly expressed when the two homologous copies are paired than when pairing is prevented by chromosome rearrangements [41]. It could be argued that the functional advantages of co-localization constitute one of the reasons why many PcG target genes are found in genomic clusters [33].

## Methods

### Transgene construction

The *LacO-Mcp* construct was described in [17]. To create the related Mcp-Eye plasmids, the eye enhancer and 820 bp *Mcp* fragments were PCR amplified using primers with appropriate restriction sites. The eye enhancer used is a 1110 bp *Hinc*II-*BanH*I fragment described in [13], and was PCR-amplified from *Drosophila* larva genomic DNA. The 820 bp *Mcp* was the widely used *Sal*I-*Xba*I fragment, amplified from BX-C clone BAC R24L18 (obtained from BACPAC Resources Center, http://bacpac.chori.org/). The amplified fragments were ligated into plasmids containing LoxP and FRT cassettes and the resulting plasmids were sequenced to verify the inserted sequence. The resulting plasmids were cut with *KpnI*, releasing the Lox-Eye En-Lox-FRT-*Mcp*-FRT fragment, which was inserted in either orientation into the acceptor plasmid containing the *mini-white* gene, yielding products with different arrangements of *Mcp* and eye enhancer relative to *mini-white*. The tandem array of 128 copies of *LacO* was isolated from pAFS150 (a gift from J. Vazquez) and inserted into the pC4Yellow vector [34] and the resulting plasmid was used to accept the *LoxP*-flanked *Mcp* insulator part, the FRT-flanked *Mcp* PRE part and *mini-white* gene.

The plasmids *Mcp*ΔPRE-Eye, *Mcp*-UAS and *Mcp-Ubx* were similarly constructed, except using different PCR-amplified fragments. The insulator part of *Mcp* used in *Mcp*ΔPRE-Eye was amplified from BX-C clone BAC R24L18, the 5×UAS fragment was amplified from pUASTattB (GenBank: EF362409.1), and the 2250 bp *Ubx* imaginal enhancer fragment was previously described [31].

### Fly stocks and genetic crosses

Transgenic fly lines were made according to standard procedures [42]. Southern blot hybridization was used to verify that the lines contained a single insert and inverse PCR was used to identify the exact insertion sites. The various deletion derivatives were established with the help of Flipase and Cre recombinase-producing stocks [43] and were verified by PCR analysis. For co-localization studies, two transgene lines on different chromosomes were crossed together using double balancers. Insertions on the same chromosome were recombined to obtain a cis-arrangement. PCR was used to verify the presence of both transgenes.

The fly line expressing EGFP-*LacI* from the ubiquitous *Ubiquitin* promoter was described in [17]. The mRFP-*LacI* fly line was kindly provided by Dr. A. Csink [44]. Mutations *Pc* ^3^ and *trx* ^E2^ are loss of function mutations (FlyBase, http://flybase.org/). Mutants *CTCF* ^y+2^, *CP190* ^p11^, *CP190* ^4-1^, *CP190* ^H31-2^, *AGO2* ^v966m^, *AGO2* ^51B^, *piwi* ^1^, *piwi* ^2^, *aub* ^QC42^, *aub* ^p-3a^, *Rm62* ^sh(3)029^, *Rm62* ^01086^ were generously provided by Drs. E.P. Lei and V.G. Corces. Mutants *Smc1* ^7-13a^, *Smc1* ^ex46^, *Rad21* ^ex15^, and *Rad21* ^36RipP^ were kindly provided by Dr. D. Dorsett. Experiments with mutations other than *trx* and *Pc* were done using trans-heterozygous allele combinations.

### 3C assay

3C experiments were done as previously described [45], [46] with few modifications, using chromatin isolated from eye and/or wing imaginal discs dissected from 100∼150 third instar larvae in 1×PBS buffer (137 mM NaCl, 2.7 mM KCl, 10 mM Na_2_HPO_4_, 2 mM KH_2_PO_4_, pH 7.4) containing 10% fetal calf serum. The tissue was fixed 10 min in 2% paraformaldehyde/PBS at room temperature. The cells were lysed in lysis buffer (10 mM Tris-HCl, 10 mM NaCl, 0.2% NP-40, pH 8.0, with Roche protease inhibitor cocktail freshly added) on ice for 10 min, followed by 20 strokes of a Dounce homogenizer. The nuclei were recovered, washed and resuspended in 400 µl 1.2×NEB3 buffer (120 mM NaCl, 60 mM Tris-HCl, 12 mM MgCl_2_, 1.2 mM Dithiothreitol, pH 7.9) with 0.3% Sodium dodecyl sulfate (SDS). After shaking for 1 hr at 37°C, Triton X-100 was added to 1.8%, and shaking was continued for another 1 hr at 37°C. After digestion with *EcoR*I (200 units) overnight, the enzyme was inactivated with 1.5% SDS at 65°C for 25 min. 1% Triton X-100 was used to neutralize the SDS at 37°C for 1 hr. The DNA was ligated in 2.4 ml with 20 µl ligase (400 U/µl, NEB) at 16°C for 4.5 hrs, then 1 hr at room temperature. The 3C template DNA was then un-crosslinked overnight at 65°C, extracted with phenol-chloroform, and dissolved in 100 µl Tris buffer (10 mM Tris·Cl).

3C primers were designed for the regions flanking the religated restriction sites, close to the insertion sites of transgenes. As a control for the crosslinking and ligation procedure we used Primers K1 and K2, close to adjacent *EcoR*I fragments in the *Brk* gene (X chromosome) and pointing in the same direction. All 3C primers are listed in Table S1. The 3C PCR reactions were done using the following cycles: denature at 95°C for 8 min, then 40 cycles of 95°C 15 s, 55°C 20 s, 72°C 20 s, finally 72°C extension for 10 min. For the K1/K2 control reactions, 36 cycles of PCR were used. To quantify the 3C interactions in BG3 and Sg4 cells, Taqman Probes (from Integrated DNA Technologies, Inc.; sequences listed in Table S1) were used for qPCRs. All 3C PCRs were repeated independently 2 or 3 times.

### Live-imaging microscopy

The live-imaging was done as described previously [17], [18]. To visualize the transgene tagged with 128 copies of LacO repeats inserted in the genome, the transgenic flies were crossed to LacI-EGFP flies, and the resultant embryos grown at 18°C in medium supplemented with active dry yeast. Third instar larvae were rinsed and dissected in Gibco Schneider's *Drosophila* medium (Invitrogen Co.). The dissected eye and wing imaginal discs were aligned on a coverslip bottom dish (MatTek Co.) with a drop of *Drosophila* medium and then covered with a coverslip. In similar imaging conditions, tissue cells have been found to stay alive for up to 12 hours. Usually, the dissected tissues were immediately subjected to direct microscopy, which finished within 1 hour. Z-stack images across at least one layer of cells were taken with a DeltaVision Image Restoration Microscope system (Applied Precision Instrument, LLC Issaquah, WA) using a 100×/1.35 UplanApo objective, deconvoluted and processed with the SoftWoRx software (Applied Precision Instruments). Each tissue in the dish was imaged no more than twice to avoid photo-bleaching. We mainly imaged eye imaginal discs and wing imaginal discs. For eye discs, only the region posterior to the morphogenetic furrow was examined. The dots in each nucleus were manually scored by moving the 3D images up and down along z-axis, one dot as co-localization and two non-overlapping dots (center-to-center distance greater than 0.3 µm) as no co-localization.

Since the expression of LacI-EGFP was driven by the Ubiquitin promoter, all the cells in all tissues under investigation showed one bright GFP dot per cell containing a single inserted transgene, showing that transgene detection is 100%. Since the LacI-EGFP contains a nuclear localization signal, a weak diffuse GFP signal demarcates each nucleus. In lines lacking *Mcp*, more than 99% of the nuclei have two dots, arguing that we are not likely to overlook one of the two transgenes [17]. In addition, when two dots are seen, the intensity of each is always lower than when a single dot is seen, indicating that the single dot is the sum of two transgene signals.

### Statistical analysis

The percentage of one-dot cells over total cells was used to measure the frequency of co-localization for each individual fly line. More than a dozen eye or wing discs were counted for each specific fly line. Variation in the percentages among eye discs or among wing discs in a given line was less than 2%. We therefore added the counts from a given tissue to represent each fly line by more than 500 cells for the eye discs or wing discs. χ^2^ tests were used for comparison of each fly line and its derivative lines or mutant backgrounds to calculate the probability that the differences might be due to chance. All statistical analysis was done with the software JMP (SAS Institute Inc.) and the results are tabulated in Table S2. Analysis of the immunofluorescence results is described under Immunofluorescence.

### Immunofluorescence

Eye and wing imaginal discs were dissected from third instar larvae and fixed with 2% para-formaldehyde for 20 minutes at room temperature. The fixed tissues were subjected to extensive washing with PBTr (1×PBS, 0.3% Triton-X100), then incubated with blocking buffer and mouse monoclonal antibody against RNA Pol II large subunit, clone 3 (generously provided by H. Saumweber) or with mouse monoclonal anti-PSC (Santa Cruz). After extensive wash, the tissue was stained with anti-mouse Cy3 secondary antibody. The slides were sealed with VECTASHIELD mounting medium (Vector Laboratories) and image acquisition was done at 100× magnification using the DeltaVision Image Restoration Microscope system. NIH ImageJ software was used to analyse the images. The 3D stack was first projected, and the background fluorescence was subtracted by adjusting the threshold. The particle size was set at >2 µm^2^, the number of bodies and the mean intensities of the bodies were computed by the software. The maximum intensities in each nucleus were also measured. The Pooled *t*-test (assuming equal variances), and Satterthwaite *t*-test (assuming unequal variances) were used to compare the difference in the number of bodies, the mean intensities, and the maximum intensities of the two sets of data (LacZ control and CTCF RNAi cells) using SAS software (SAS Institute Inc.). The two *t*-tests gave similar results and box plots were used to present the data, with the median shown as a line across the box and the mean indicated by “+”.

### Cell culture and RNAi

Drosophila ML-DmBG3-c2 cells (abbreviated: BG3) cells and Sg4 cells were cultured in Schneider's Drosophila Medium (Invitrogen) supplemented with 10% heat-inactivated fetal bovine serum. Double-stranded RNAs for CTCF and for lacZ as a negative control were prepared according to the user's manual using the RiboMAX Large Scale RNA Production System—T7 (Promega). Genomic DNA was used to amplify the template for dsRNA synthesis. The primers are listed in Table S1. The RNAi procedure exactly followed ref. [47]. After RNAi treatments, the harvested cells were lysed and subjected to western blots (Figure S5) to verify the efficacy with mouse anti-CTCF antibody (generously provided by V.G. Corces). The RNAi knockdown cells were fixed with 2% paraformaldehyde, and then either subjected to the 3C procedure with 10^7^ cells, or used for the immunofluorescence experiments as described above.

## Supporting Information

Figure S1 Eye color phenotypes and co-localization effects of the Eye enhancer. (A) Eye colors of the *Mcp*-Eye fly lines. Three *Mcp*-Eye-B lines all show pairing-sensitive silencing of the *mini-white* gene, while *Mcp*-Eye-A22 flies do not. In each image, the head on the left is heterozygous for the transgene insertion while the head on the right is homozygous but has lower expression of the *white* gene and lower eye pigmentation. (B) Single enhancer deletion has similar effects on co-localization of two transgenes as double enhancer deletions. Three *Mcp*-Eye transgene pairs were tested by live-imaging either intact, or with the enhancer deleted in one transgene, or with the enhancers deleted in both. As a negative control, deletion of both *Mcp*s was also tested.
(TIF)

Figure S2 Effects of RNAi and cohesin mutations on co-localization. (A) The effects of co-localization of a pair of *Mcp*-Eye insertions (intact) after deletion of the eye enhancer (ΔE), of *Mcp* (ΔM) or with loss of function mutations of *Rm62*, *piwi* or *aub*. AGO2^V966M^, a mutation in the AGO2 catalytic site, has no effect but the loss of function allele AGO2^51B^ reduces high level co-localization to the basal level. (B) Effects of cohesin mutations. Co-localization is not affected by heteroallelic loss of function mutations *Smc1* ^7-13a^/*Smc1* ^ex46^ or *Rad21* ^ex15^/*Rad21* ^36RipP^.
(TIF)

Figure S3 Co-localization of *Mcp*-Eye with Polycomb bodies or transcription factories. (A) The transgene labeled with *LacI*-RFP (red) was visualized in eye or wing discs of flies expressing PC-GFP (green) and in (B) the transgene was labeled with *LacI*-EGFP (green) and the imaginal discs were stained with anti-RNA pol II (red). The transgene (arrows indicate typical co-localization examples) associates with PC in ∼20% of wing nuclei but in <1% of eye nuclei (arrowheads indicate typical no co-localization examples). It associates with RNA pol II in ∼10% of wing nuclei (arrowheads show typical no co-localization) but in 80% of eye nuclei (arrows show the co-localization).
(TIF)

Figure S4 Arm-Gal4/UAS enhancer can also promote co-localization both in the eye and wing disc cells. The upper panel shows the map of *Mcp*-UAS containing 5 GAL4 consensus sequences, and lower panel shows the co-localization of pairs of *Mcp*-UAS insertions in the presence of the *LacI*-EGFP alone or of *LacI*-GFP plus the Arm-GAL4 driver to activate transgene expression. A weak but significant increase in co-localization is observed in both eye and wing discs, consistent with the ubiquitous expression of *Arm*-GAL4.
(TIF)

Figure S5 CTCF, TRX and ASH1 RNAi treatments knock down the target proteins in both Bg3 and Sg4 cell cultures. The cells were treated either with *LacZ* dsRNA (control) or CTCF dsRNA, or TRX dsRNA, or ASH1 dsRNA for three rounds, then lysed and extracts were subjected to western blot with α-CTCF antibody (A), or α-TRX antibody (B), or α-ASH1 antibody (C), β-Tubulin was used as loading control. (D) CTCF knockdown does not affect the expression level of PSC. BG3 cells were treated with either LacZ dsRNA (as control) or CTCF dsRNA for three rounds, then lysed and extracts were subjected to western blot with α-PSC antibody, β-Tubulin was used as loading control.
(JPG)

Figure S6 CTCF knockdown abrogates the long-range interaction detected by 3C between *Abd-B* and *Antp* genes, which are both PcG-repressed in BG3 cells. The CTCF dependence of this emblematic Polycomb body interaction implies that those Polycomb bodies that are formed by the association of remote PcG targets would fall apart in the absence of CTCF.
(TIF)

Figure S7 Effect of CTCF RNAi on transcription factories. A monoclonal antibody against RNA polymerase II large subunit was used to illuminate the transcription factories in both LacZ and CTCF RNAi treated Bg3 cells (red). DNA was stained with DAPI (blue). CTCF knockdown does not have major effects on the number or intensity of the transcription factories.
(TIF)

Table S1 List of PCR primers.
(DOC)

Table S2 Statistical analysis.
(DOC)

Video S1 Z-stack scan of co-localization in the eye imaginal disc. The 3^rd^ instar larva eye imaginal disc eye columnar cells were imaged under wide-field fluorescence microscope. The images were taken along the z-axis of the tissue every 0.3 µm, then reconstituted into a 3D movie, and the dots in each nucleus were counted. The movie shown here shows typical *Mcp*-Eye-B4–*Mcp*-Eye-B15 fly line eye columnar cell clusters. Notice that the eye membrane cells could be seen at the beginning of the movie and appear always with two dots in each nucleus, while the ommatidial columnar (photoreceptor) cells mostly (∼86%) have only one dot.
(WMV)
