# Supplementary figures and images for: Insulators Target Active Genes to Transcription Factories and Polycomb-Repressed Genes to Polycomb Bodies
Source: PLoS Genet. 2013 Apr 18;9(4):e1003436. doi: 10.1371/journal.pgen.1003436 (PMC3630138; doi:10.1371/journal.pgen.1003436)

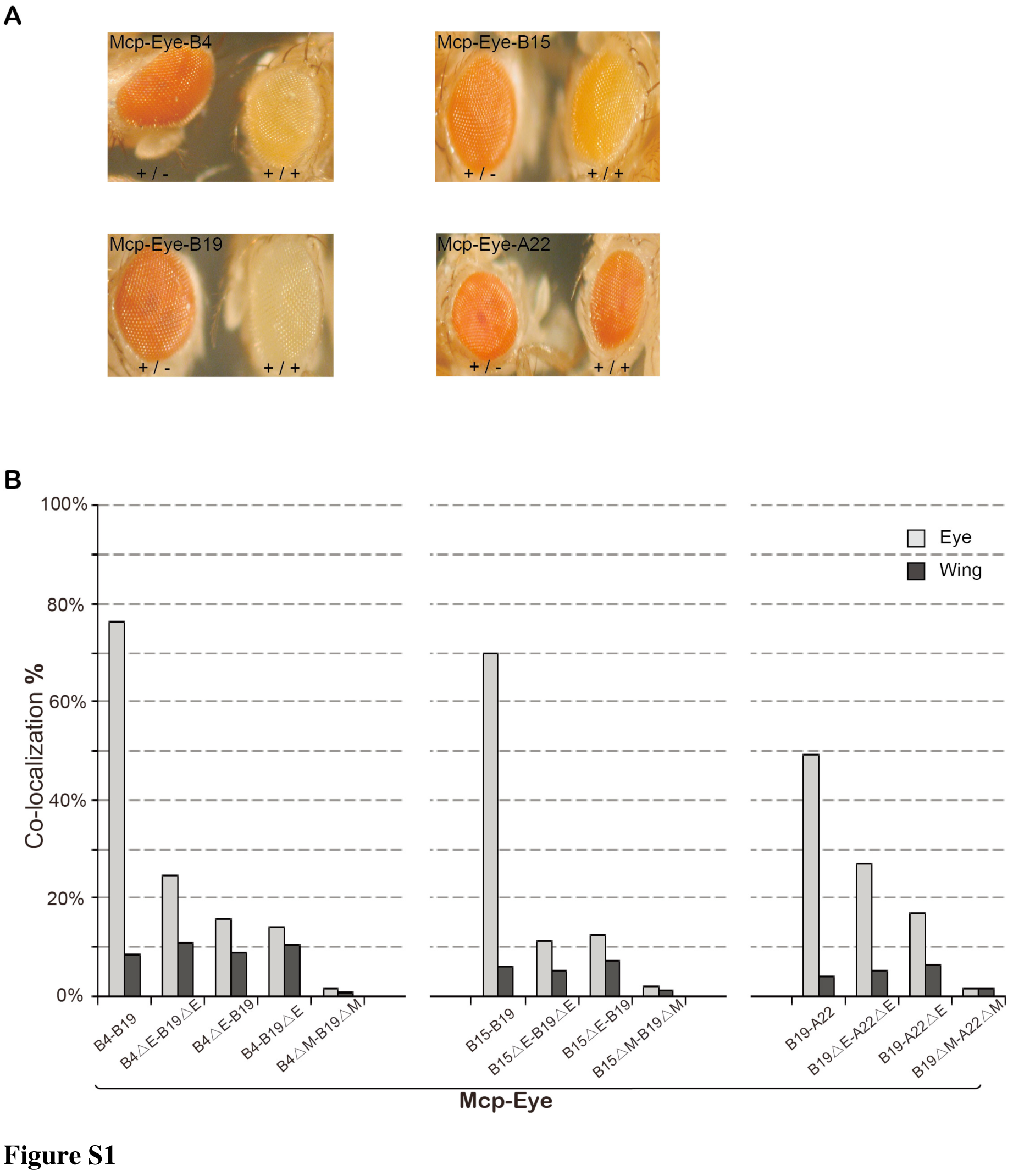

Supplement: Figure S1 — Eye color phenotypes and co-localization effects of the Eye enhancer. (A) Eye colors of the Mcp-Eye fly lines. Three Mcp-Eye-B lines all show pairing-sensitive silencing of the mini-white gene, while Mcp-Eye-A22 flies do not. In each image, the head on the left is heterozygous for the transgene insertion while the head on the right is homozygous but has lower expression of the white gene and lower eye pigmentation. (B) Single enhancer deletion has similar effects on co-localization of two transgenes as double enhancer deletions. Three Mcp-Eye transgene pairs were tested by live-imaging either intact, or with the enhancer deleted in one transgene, or with the enhancers deleted in both. As a negative control, deletion of both Mcps was also tested. (TIF) [file pgen.1003436.s001.tif]

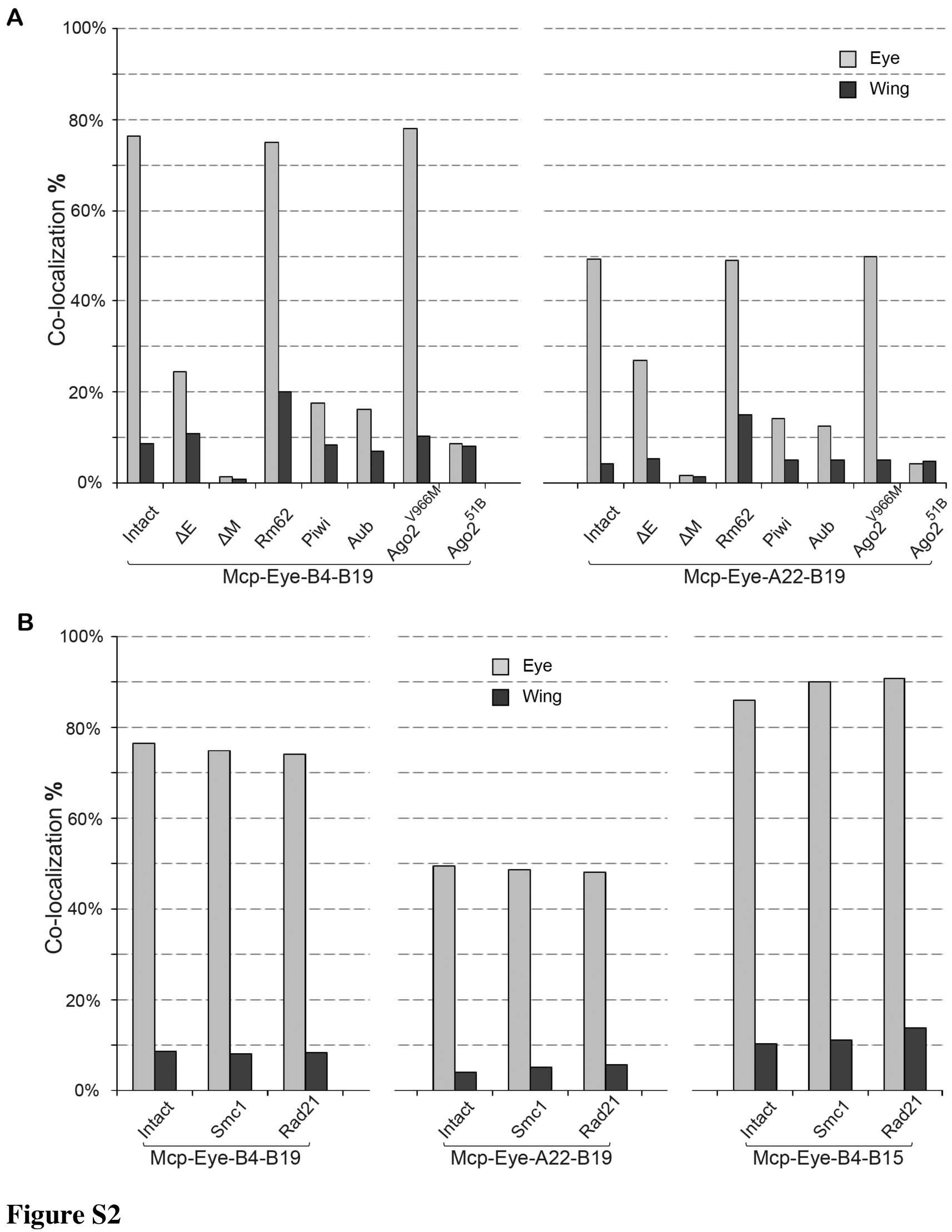

Supplement: Figure S2 — Effects of RNAi and cohesin mutations on co-localization. (A) The effects of co-localization of a pair of Mcp-Eye insertions (intact) after deletion of the eye enhancer (ΔE), of Mcp (ΔM) or with loss of function mutations of Rm62, piwi or aub. AGO2V966M, a mutation in the AGO2 catalytic site, has no effect but the loss of function allele AGO251B reduces high level co-localization to the basal level. (B) Effects of cohesin mutations. Co-localization is not affected by heteroallelic loss of function mutations Smc1 7-13a/Smc1 ex46 or Rad21 ex15/Rad21 36RipP. (TIF) [file pgen.1003436.s002.tif]

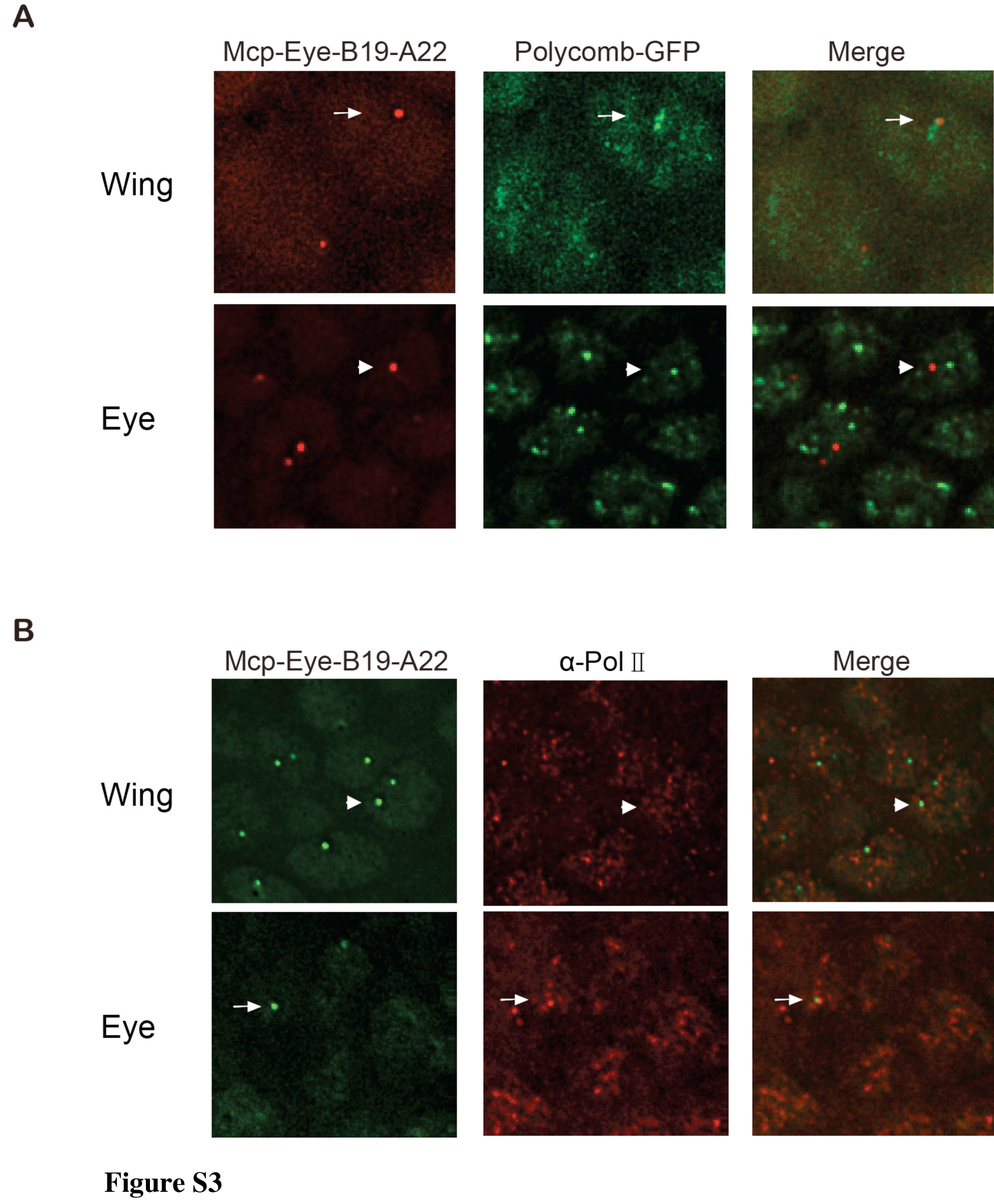

Supplement: Figure S3 — Co-localization of Mcp-Eye with Polycomb bodies or transcription factories. (A) The transgene labeled with LacI-RFP (red) was visualized in eye or wing discs of flies expressing PC-GFP (green) and in (B) the transgene was labeled with LacI-EGFP (green) and the imaginal discs were stained with anti-RNA pol II (red). The transgene (arrows indicate typical co-localization examples) associates with PC in ∼20% of wing nuclei but in <1% of eye nuclei (arrowheads indicate typical no co-localization examples). It associates with RNA pol II in ∼10% of wing nuclei (arrowheads show typical no co-localization) but in 80% of eye nuclei (arrows show the co-localization). (TIF) [file pgen.1003436.s003.tif]

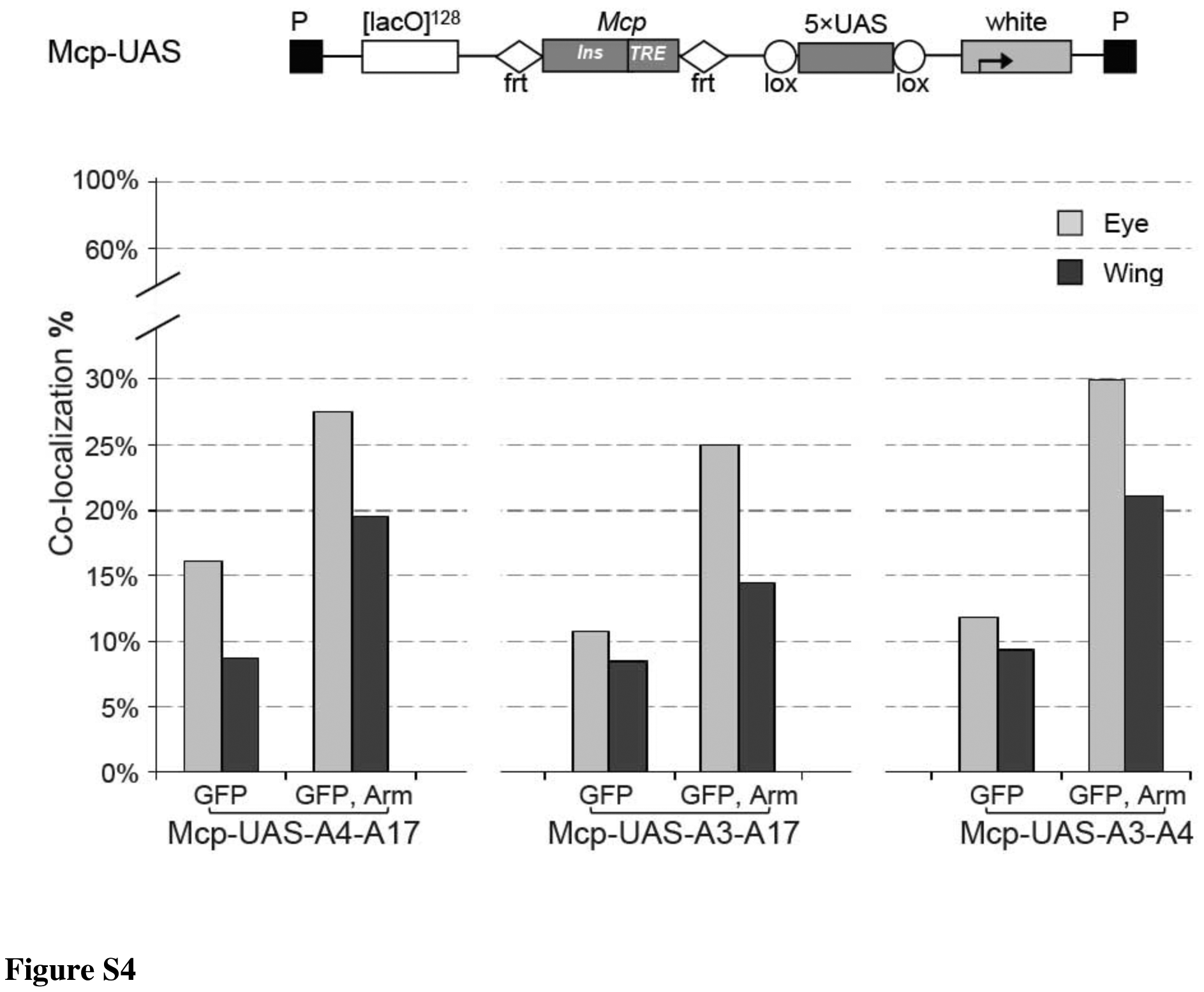

Supplement: Figure S4 — Arm-Gal4/UAS enhancer can also promote co-localization both in the eye and wing disc cells. The upper panel shows the map of Mcp-UAS containing 5 GAL4 consensus sequences, and lower panel shows the co-localization of pairs of Mcp-UAS insertions in the presence of the LacI-EGFP alone or of LacI-GFP plus the Arm-GAL4 driver to activate transgene expression. A weak but significant increase in co-localization is observed in both eye and wing discs, consistent with the ubiquitous expression of Arm-GAL4. (TIF) [file pgen.1003436.s004.tif]

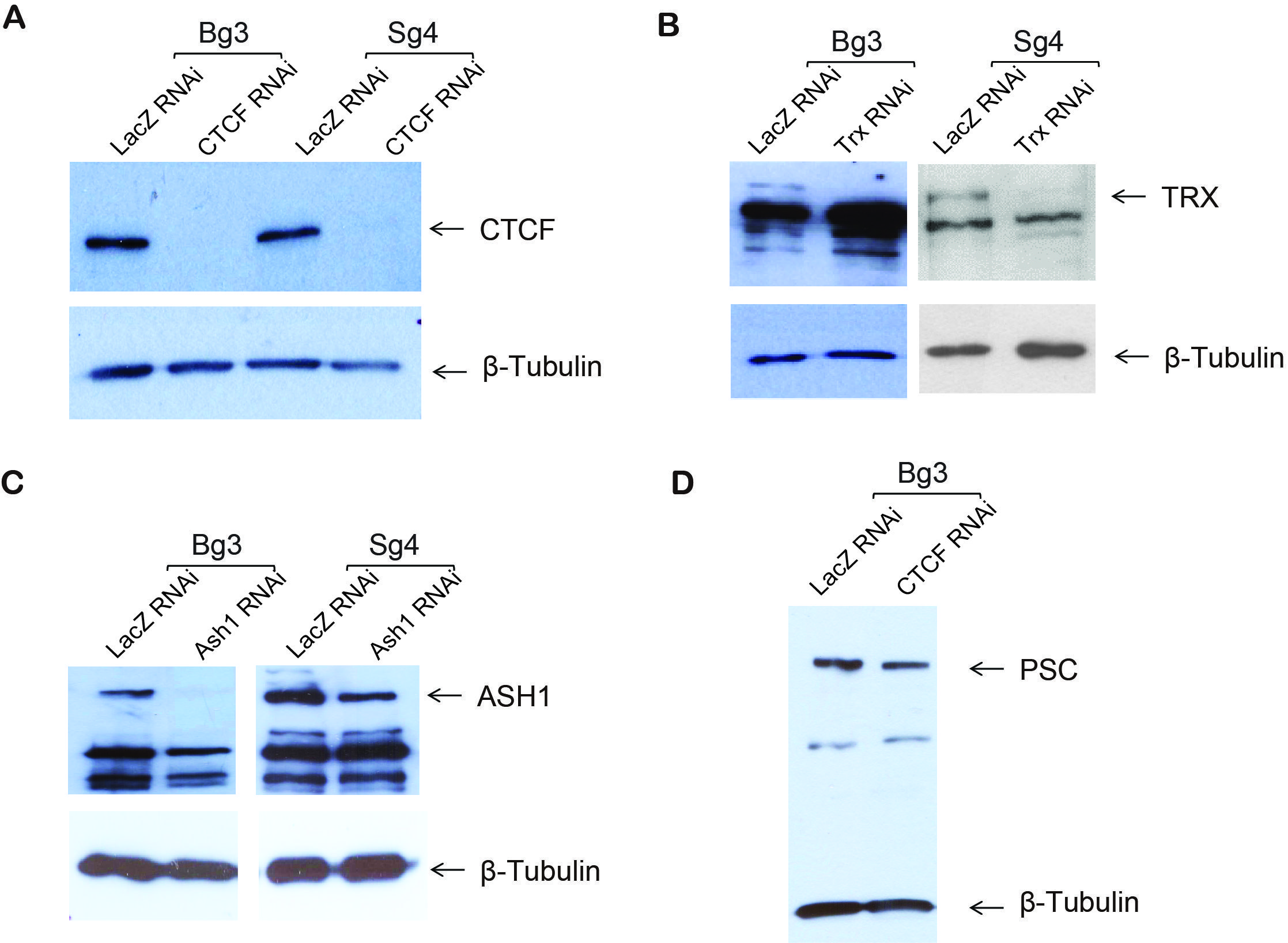

Supplement: Figure S5 — CTCF, TRX and ASH1 RNAi treatments knock down the target proteins in both Bg3 and Sg4 cell cultures. The cells were treated either with LacZ dsRNA (control) or CTCF dsRNA, or TRX dsRNA, or ASH1 dsRNA for three rounds, then lysed and extracts were subjected to western blot with α-CTCF antibody (A), or α-TRX antibody (B), or α-ASH1 antibody (C), β-Tubulin was used as loading control. (D) CTCF knockdown does not affect the expression level of PSC. BG3 cells were treated with either LacZ dsRNA (as control) or CTCF dsRNA for three rounds, then lysed and extracts were subjected to western blot with α-PSC antibody, β-Tubulin was used as loading control. (JPG) [file pgen.1003436.s005.jpg]

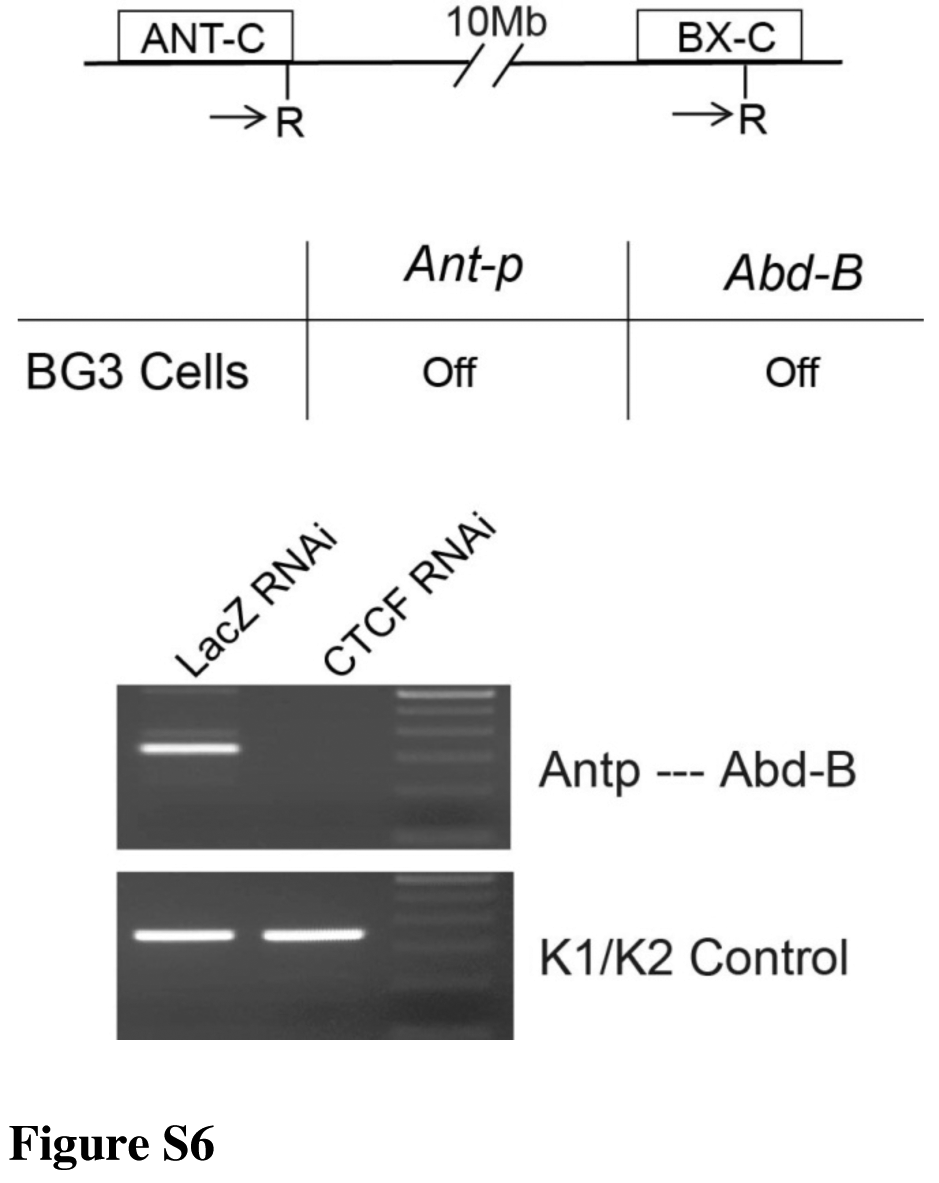

Supplement: Figure S6 — CTCF knockdown abrogates the long-range interaction detected by 3C between Abd-B and Antp genes, which are both PcG-repressed in BG3 cells. The CTCF dependence of this emblematic Polycomb body interaction implies that those Polycomb bodies that are formed by the association of remote PcG targets would fall apart in the absence of CTCF. (TIF) [file pgen.1003436.s006.tif]

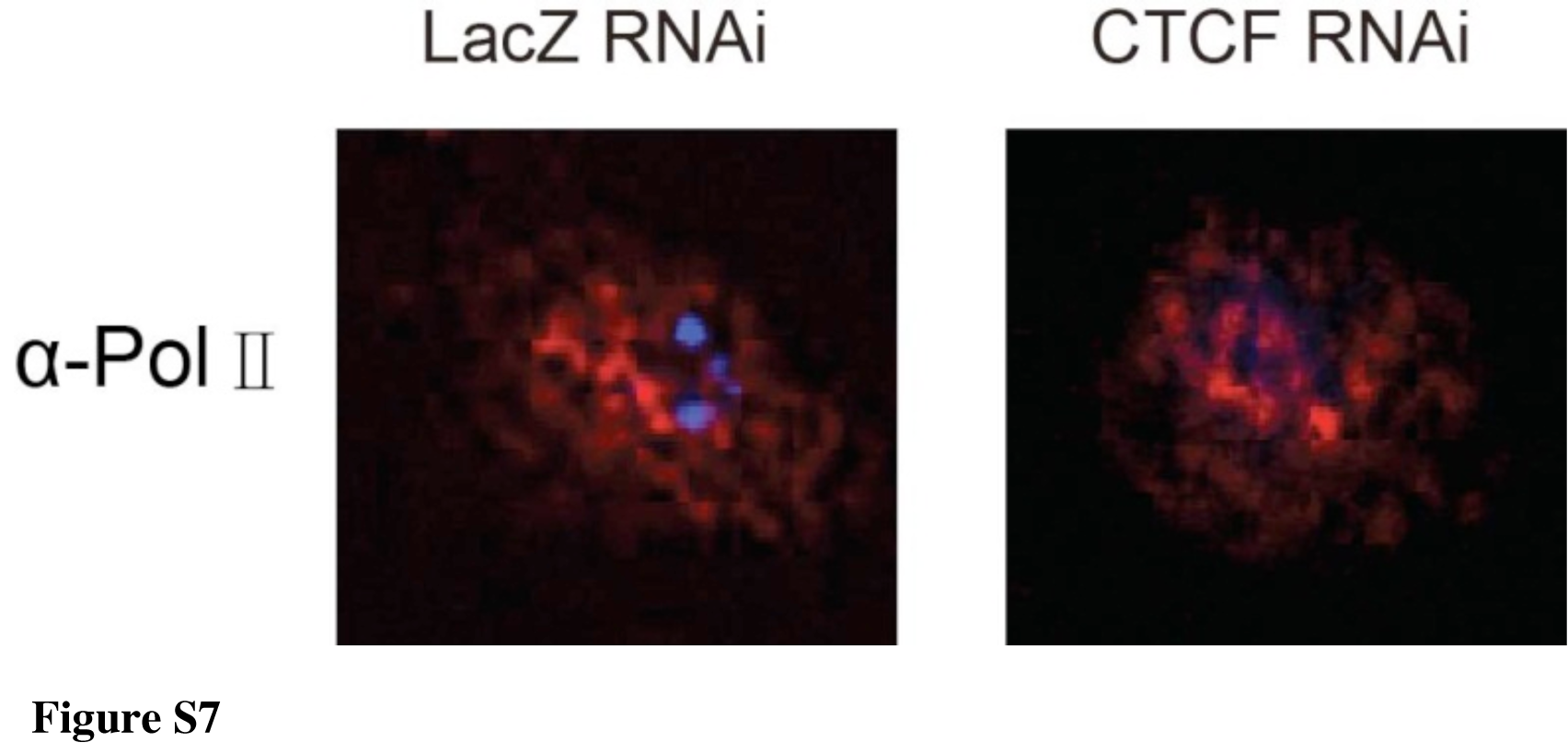

Supplement: Figure S7 — Effect of CTCF RNAi on transcription factories. A monoclonal antibody against RNA polymerase II large subunit was used to illuminate the transcription factories in both LacZ and CTCF RNAi treated Bg3 cells (red). DNA was stained with DAPI (blue). CTCF knockdown does not have major effects on the number or intensity of the transcription factories. (TIF) [file pgen.1003436.s007.tif]
