# Supplementary material for: Insulators Target Active Genes to Transcription Factories and Polycomb-Repressed Genes to Polycomb Bodies
Source: PLoS Genet. 2013 Apr 18;9(4):e1003436. doi: 10.1371/journal.pgen.1003436 (PMC3630138; doi:10.1371/journal.pgen.1003436)
Supplement: Table S1 — List of PCR primers. (DOC) [file pgen.1003436.s008.doc]

**Table S1 List of PCR primers**

| **Amplicon name** | **Amplicon length, bp** | **Primer name** | **Sequence 5’ to 3’** |
| --- | --- | --- | --- |
| White Enhancer | 1130 | we1 | ctgg GAATTC agtcaacccagaccaacc |
|  |  | we2 | gatatggatccgactgggacg |
| Mcp | 820 | Mcp+ | ctggGGATCCggccgttttccgttttattg |
|  |  | M820- | cgaaGCATGCtagaaaaattccgcaccag |
| *Mcp*DPRE | 530 | Mcp+ | ctggGGATCCggccgttttccgttttattg |
|  |  | M530- | cactGCATGC tgagaaacccaagcgttg |
| 5×UAS | 200 | Gal4+ | cttgGAGCTCCGCGGcactggaactaggctag |
|  |  | Gal4- | cgttGAATTCcggcgctcgctagag |
| Ubx Enhancer | 2250 | ubxE1 | ctaaGAATTCtaccgcttgtaaccgctatg |
|  |  | ubxE2 | gcaaGGATCCtgcccgacaaactttactaac |
|  | | | |
| 3C Control | 357 | K1 | CACGGGAAAAACTACTGAAAG |
|  |  | K2 | AAGCCGCAGGAGTTTCTAAC |
|  |  | Brk-probe | /56-FAM/CACCGAAGA/ZEN/CGAGACCGTTGAC /3IABkFQ |
| Mcp-Eye-B4---B19 | 114 | B4 | GCCAAAAGCCCAAGAAGGTG |
|  |  | B19 | CAGTAACCATTGCCATTACAC |
| Mcp-Ubx-A---B | 198 | A | GTCGTTCGCCTTTTTTGTTTG |
|  |  | B1 | CAGCGTTGTACCAAATTAGAC |
| Mcp---Mcp-Ubx-A | 426 | A | GTCGTTCGCCTTTTTTGTTTG |
|  |  | M5 | GCATGGCGGCATAATTTCTG |
| Mcp---Mcp-Ubx-B | 257 | B2 | CTCCATGACCATTTGCTGTTG |
|  |  | M5.2 | GCCTTCGTTGAGCACAAAAC |
| ANTC---Mcp-Ubx-A | 314 | A | GTCGTTCGCCTTTTTTGTTTG |
|  |  | ANTC | GGTCGGGCTCTGTTTGTATG |
| ANTC---Mcp-Ubx-B | 256 | B2 | CTCCATGACCATTTGCTGTTG |
|  |  | ANTC | GGTCGGGCTCTGTTTGTATG |
| Antp---Mcp-Ubx-B | 273 | B1 | CAGCGTTGTACCAAATTAGAC |
|  |  | Antp | ACGAATGGATGGCAACAGTG |
| Dfd---Mcp-Ubx-B | 204 | B2 | CTCCATGACCATTTGCTGTTG |
|  |  | Dfd | ACCACCCCACACCCATTTG |
| Pnt---c15 | 115bp | Pnt2d | AACAAGTGGGAACGGAACTC |
|  |  | NK4d | CCACGCTCCGTCCAAAAAG |
|  |  | Pnt-probe | /56-FAM/CTGGAAACC/ZEN/CTCTTGCGGAACG  /3IABkFQ/ |
| Abd-B---lbe | 201bp | Fab3C2 | GAAGTCGGGAGGCAAAACTA |
|  |  | NK2u | CAATGAACGCCCACCACTC |
|  |  | Fab-probe | /56-FAM/ACATCAGGA/ZEN/AGAGGTTCGTTGGA  /3IABkFQ/ |
| Abd-B---pnt | 183bp | Fab3C2 | GAAGTCGGGAGGCAAAACTA |
|  |  | Pnt2d | AACAAGTGGGAACGGAACTC |
|  |  | Pnt-probe | /56-FAM/CTGGAAACC/ZEN/CTCTTGCGGAACG  /3IABkFQ/ |
| Abd-B---c15 | 186bp | Fab3C2 | GAAGTCGGGAGGCAAAACTA |
|  |  | NK4d | CCACGCTCCGTCCAAAAAG |
|  |  | Fab-probe | /56-FAM/ACATCAGGA/ZEN/AGAGGTTCGTTGGA /3IABkFQ/ |
| ANTC---AbdB | 330 | ANTC | GGTCGGGCTCTGTTTGTATG |
|  |  | Fab | GGATGAACGGAAGATGTCTG |
|  | | | |
| LacZ RNAi | 683bp | Lac-F | CTAATACGACTCACTATAGGGAGGGCCACCGATATTATTTGCC |
|  |  | Lac-R | CTAATACGACTCACTATAGGGAGCACACTGAGGTTTTCCGC |
| CTCF RNAi | 587bp | CTCF-F | CTAATACGACTCACTATAGGGAGAACGAGGCTGAGGTCTACGA |
|  |  | CTCF-R | CTAATACGACTCACTATAGGGAGTAAATCGCGACTTGCAGATG |
| Trx RNAi | 839bp | Trx-F | CTAATACGACTCACTATAGGGAGCAAACGTCTACCACCACCC |
|  |  | Trx-R | CTAATACGACTCACTATAGGGAGGCCCATTAGCGTGGTATCC |
| Ash1 RNAi | 1037bp | Ash1-F | CTAATACGACTCACTATAGGGAGgcagtgccatggagaccc |
|  |  | Ash1-R | CTAATACGACTCACTATAGGGAGcaacacccagcagcgtcc |
