# Supplementary material for: Insulators Target Active Genes to Transcription Factories and Polycomb-Repressed Genes to Polycomb Bodies
Source: PLoS Genet. 2013 Apr 18;9(4):e1003436. doi: 10.1371/journal.pgen.1003436 (PMC3630138; doi:10.1371/journal.pgen.1003436)
Supplement: Table S2 — Statistical analysis. (DOC) [file pgen.1003436.s009.doc]

**Table S2 Statistical analysis.**

|  | **Percentage of Colocalization** | **Total Cell Number** | **CHI-SQUARE (χ²)** | **p-value** |
| --- | --- | --- | --- | --- |
| **Eye cells of Mcp-Eye fly lines** | | | | |
| **B4-B19** | 76.40% | 644 | 308.68 | <.0001 |
| B4∆E-B19∆E | 24.60% | 516 |
|  | | | | |
| B4-B19 | 76.40% | 644 | 726.49 | <.0001 |
| B4∆M-B19∆M | 1.40% | 628 |
|  | | | | |
| B4-B19 | 76.40% | 644 | 1277.37 | <.0001 |
| B4∆M∆E-B19∆M∆E | 0.19% | 1245 |
|  | | | | |
| B4-B19 | 76.40% | 644 | 1243.04 | <.0001 |
| B4-B19; CTCF ^y+2^ | 0.80% | 1245 |
|  | | | | |
| B4-B19 | 76.40% | 644 | 514.702 | <.0001 |
| B4-B19;CP190 ^p11/4-1^ | 14.90% | 700 |
|  | | | | |
| B4-B19 | 76.40% | 644 | 663.048 | <.0001 |
| B4-B19; CP190 ^p11/H31-2^ | 13.00% | 980 |
|  | | | | |
| B4-B19 | 76.40% | 664 | 0.0001 | 0.9913 |
| B4-B19; Pc^3^ | 76.30% | 845 |
|  | | | | |
| B4-B19 | 76.40% | 664 | 589.11 | <.0001 |
| B4-B19; Trx^1^ | 8.40% | 597 |
|  | | | | |
| B4-B19 | 76.40% | 644 | 0.37 | 0.543 |
| B4-B19; Rm62^sh3029/E^ | 75.00% | 841 |
|  | | | | |
| B4-B19 | 76.40% | 644 | 495.35 | <.0001 |
| B4-B19; PIWI^1/2^ | 17.40% | 774 |
|  | | | | |
| B4-B19 | 76.40% | 644 | 348.23 | <.0001 |
| B4-B19; Aub^QC42/∆p-3a^ | 16.00% | 375 |
|  | | | | |
| B4-B19 | 76.40% | 644 | 0.498 | 0.48 |
| B4-B19; AGO2^V966M^ | 78.00% | 687 |
|  | | | | |
| B4-B19 | 76.40% | 644 | 558.49 | <.0001 |
| B4-B19; AGO2^51B^ | 8.50% | 558 |
|  | | | | |
| B4-B19 | 76.40% | 644 | 0.974 | 0.3238 |
| B4-B19; Rad21^36RipP/ex15^ | 74.00% | 698 |
|  | | | | |
| B4-B19 | 76.40% | 644 | 0.368 | 0.544 |
| B4-B19; Smc1^7-13a/ex46^ | 75.00% | 752 |
|  | | | | |
| **A22-B19** | 49.40% | 503 | 61.6 | <.0001 |
| A22∆E-B19∆E | 26.90% | 664 |
|  | | | | |
| A22-B19 | 49.40% | 503 | 425.74 | <.0001 |
| A22∆M-B19∆M | 1.70% | 775 |
|  | | | | |
| A22-B19 | 49.40% | 503 | 629.18 | <.0001 |
| A22∆M∆E-B19∆M∆E | 1.24% | 1205 |
|  | | | | |
| A22-B19 | 49.40% | 503 | 411.402 | <.0001 |
| A22-B19; CTCF ^y+2^ | 0.60% | 684 |
|  | | | | |
| A22-B19 | 49.40% | 503 | 199.78 | <.0001 |
| A22-B19;CP190 ^p11/4-1^ | 13.70% | 824 |
|  | | | | |
| A22-B19 | 49.40% | 503 | 197.01 | <.0001 |
| A22-B19; CP190 ^p11/H31-2^ | 12.00% | 665 |
|  | | | | |
| A22-B19 | 49.40% | 503 | 0.177 | 0.674 |
| A22-B19; Pc^3^ | 48.10% | 852 |
|  | | | | |
| A22-B19 | 49.40% | 503 | 328.4 | <.0001 |
| A22-B19; Trx^1^ | 8.50% | 1025 |
|  | | | | |
| A22-B19 | 49.40% | 503 | 0.005 | 0.94 |
| A22-B19; Rm62^sh3029/E^ | 49.00% | 542 |
|  | | | | |
| A22-B19 | 49.40% | 503 | 199.12 | <.0001 |
| A22-B19; PIWI^1/2^ | 14.30% | 877 |
|  | | | | |
| A22-B19 | 49.40% | 503 | 188.48 | <.0001 |
| A22-B19; Aub^QC42/∆p-3a^ | 12.60% | 663 |
|  | | | | |
| A22-B19 | 49.40% | 503 | 0.058 | 0.81 |
| A22-B19; AGO2^V966M^ | 50.00% | 756 |
|  | | | | |
| A22-B19 | 49.40% | 503 | 436.32 | <.0001 |
| A22-B19; AGO2^51B^ | 4.20% | 998 |
|  | | | | |
| A22-B19 | 49.40% | 503 | 0.224 | 0.6358 |
| A22-B19; Rad21^36RipP/ex15^ | 48.00% | 865 |
|  | | | | |
| A22-B19 | 49.40% | 503 | 0.063 | 0.8022 |
| A22-B19; Smc1^7-13a/ex46^ | 48.60% | 544 |
|  | | | | |
| **B4-B15** | 85.90% | 875 | 226.63 | <.0001 |
| B4∆E-B15∆E | 51.10% | 702 |
|  | | | | |
| B4-B15 | 85.90% | 875 | 998.424 | <.0001 |
| B4∆M-B15∆M | 14.90% | 1121 |
|  | | | | |
| B4-B15 | 85.90% | 875 | 1029.85 | <.0001 |
| B4∆M∆E-B15∆M∆E | 12.40% | 1028 |
|  | | | | |
| B4-B15 | 85.90% | 875 | 1087.64 | <.0001 |
| B4-B15; CTCF^y+2^ | 10.40% | 1023 |
|  | | | | |
| B4-B15 | 85.90% | 875 | 441.94 | <.0001 |
| B4-B15; CP190 ^p11/4-1^ | 32.60% | 604 |
|  | | | | |
| B4-B15 | 85.90% | 875 | 866.28 | <.0001 |
| B4-B15; Trx^1^ | 12.50% | 741 |
|  | | | | |
| B4-B15 | 85.90% | 875 | 8.072 | 0.0045 |
| B4-B15; Rad21^36RipP/ex15^ | 91.00% | 554 |
|  | | | | |
| B4-B15 | 85.90% | 875 | 5.85 | 0.0156 |
| B4-B15; Smc1^7-13a/ex46^ | 90.00% | 689 |
|  | | | | |
| **B15-B19** | 70% | 669 | 539.73 | <.0001 |
| B15∆E-B19∆E | 11.30% | 821 |
|  | | | | |
| B15-B19 | 70% | 669 | 646.56 | <.0001 |
| B15∆M-B19∆M | 2.80% | 659 |
|  | | | | |
| B15-B19 | 70% | 669 | 954.15 | <.0001 |
| B15∆M∆E-B19∆M∆E | 0.34% | 992 |
|  | | | | |
| B15-B19 | 70.00% | 669 | 763.34 | <.0001 |
| B15-B19; CTCF^y+2^ | 0.90% | 759 |
|  | | | | |
| **A8-A22** | 71.30% | 623 | 398.73 | <.0001 |
| A8∆E-A22∆E | 13.80% | 567 |
|  | | | | |
| A8-A22 | 71.30% | 623 | 846.47 | <.0001 |
| A8∆M-A22∆M | 1.10% | 874 |
|  | | | | |
| **Eye and Wing cells of Mcp-Ubx fly lines** | | | | |
| 128A18 - B4 | 0.55 | 1560 | 316.7 | <.0001 |
| A18△E - B4△E | 0.231 | 1422 |
|  | | | | |
| 128A18 - B4 | 0.55 | 1560 | 664.33 | <.0001 |
| A18 - B4△M | 0.096 | 1350 |
|  | | | | |
| 128A18 - B4 | 0.55 | 1560 | 744.486 | <.0001 |
| A18△M - B4△M | 0.083 | 1444 |
|  | | | | |
| **Mcp-Eye fly lines + Mcp-Ubx fly lines** | | | | |
| Mcp-Eye-A22, Mcp-Ubx-B4 --- Wing | 0.099 | 123 | 85.18 | <0.0001 |
| Mcp-Eye-A22, Mcp-Ubx-B4 --- Eye | 0.901 | 1124 |
|  | | | | |
| Mcp-Eye-B4, Mcp-Ubx-B4 --- Wing | 0.088 | 101 | 76.12 | <0.0001 |
| Mcp-Eye-B4, Mcp-Ubx-B4 --- Eye | 0.912 | 1044 |
